# Cotton *BOP1* mediates SUMOylation of GhBES1 to regulate fibre development and plant architecture

**DOI:** 10.1111/pbi.14428

**Published:** 2024-07-14

**Authors:** Bingting Wang, Zhian Wang, Ye Tang, Naiqin Zhong, Jiahe Wu

**Affiliations:** ^1^ State Key Laboratory of Plant Genomics Institute of Microbiology, Chinese Academy of Sciences Beijing China; ^2^ Institute of Cotton Research, Shanxi Agricultural University Yuncheng China

**Keywords:** blade‐on‐petiole, upland cotton, fibre development, plant architecture, SUMOylation, BR signalling pathway

## Abstract

The Arabidopsis *BLADE‐ON‐PETIOLE* (*BOP*) genes are primarily known for their roles in regulating leaf and floral patterning. However, the broader functions of *BOPs* in regulating plant traits remain largely unexplored. In this study, we investigated the role of the *Gossypium hirsutum BOP1* gene in the regulation of fibre length and plant height through the brassinosteroid (BR) signalling pathway. Transgenic cotton plants overexpressing *GhBOP1* display shorter fibre lengths and reduced plant height compared to the wild type. Conversely, GhBOP1 knockdown led to increased plant height and longer fibre, indicating a connection with phenotypes influenced by the BR pathway. Our genetic evidence supports the notion that GhBOP1 regulates fibre length and plant height in a GhBES1‐dependent manner, with GhBES1 being a major transcription factor in the BR signalling pathway. Yeast two‐hybrid, luciferase complementation assay and pull‐down assay results demonstrated a direct interaction between GhBOP1 and GhSUMO1, potentially forming protein complexes with GhBES1. *In vitro* and *in vivo* SUMOylation analyses revealed that GhBOP1 functions in an E3 ligase‐like manner to mediate GhBES1 SUMOylation and subsequent degradation. Therefore, our study not only uncovers a novel mechanism of GhBES1 SUMOylation but also provides significant insights into how GhBOP1 regulates fibre length and plant height by controlling GhBES1 accumulation.

## Introduction

Cotton (*Gossypium hirsutum*) stands as the major natural textile crop globally and is also a notable source of oil, ranking second only to soybean (Wu *et al*., 2022). The architecture of the cotton plant plays a pivotal role in influencing both yield and the efficiency of mechanized harvesting (Huang *et al*., 2022). The ideal plant architecture of cotton is vital for enhancing yield and optimizing harvesting efficiency. While genes crucial for determining plant architecture in Arabidopsis have been extensively studied (Wang *et al*., 2018a), the *Gossypium* genus has been the focus of current research, identifying numerous genes and QTLs associated with cotton architecture through genome‐wide association studies (Cui *et al*., 2011; Su *et al*., 2018). Plant height is a major traits of plant architecture, the appropriate plant height could increase plant density and improve photosynthetic efficiency. Like other crops, decreased height also strengthens resistance to lodging when encounter heavy rain or high wind damage. Moreover, dwarf cotton is suitable for mechanical picking. However, it is noteworthy that the number of genes governing plant height is relatively limited. Given the significance of plant height as a key agronomic trait in crops, there is a compelling need for further research in the domain of cotton plant architecture.

*BOPs* (*Blade‐on‐petiole*) were initially identified as regulators of leaf and floral in Arabidopsis recognized as marker genes at lateral organ boundaries (Ha *et al*., 2004; Hepworth *et al*., 2005; Norberg *et al*., 2005). The BOPs contain a conserved subclade featuring BTB/POZ domains and ankyrin repeats, with the BTB domain playing a role in protein interaction and oligomerization (Stogios *et al*., 2005). *BOP* genes have ancient development function in plants. In *Physcomitrella*, PpBOP1/2 mediated the transition to reproductive development by promoting bud meristem formation (Saleh *et al*., 2011). In Arabidopsis, BOPs promoted the simple leaf shape and adaxial fate through repressing the activity of class I KNOX genes (Hay and Tsiantis, 2010). It reported that BOP1/2 repressed KNOX genes through activating ASYMMETRIC LEAVES2 (AS2) (Chalfun‐Junior *et al*., 2005). BOP1/2 also promote floral‐meristem development through activation of APETALA1(AP1). For plant architecture, gain‐of‐function studies revealed that BOP misexpression in the stem alters the length of internode elongation. In addition, it also involved in nectary development, formation of abscission zones and suppressed the formation of bract (Mckim *et al*., 2008). Due to the function of BOPs, the *bop1bop2* double T‐DNA insertion mutants exhibited elongated leafy petioles and displayed inflorescence‐like flowers with bracts and branching, indicative of defects in floral‐meristem identity (Xu *et al*., 2010). While the functions of BOPs are well‐elucidated in leaves and flowers, numerous questions remain unanswered. Despite genomic approaches identifying target genes associated with lateral organ boundaries and floral meristems, it remains unclear whether BOPs participate in hormone signalling pathways or play indirect roles in plant growth and development.

SUMOylation, a fast and potent post‐translational modification, orchestrates various aspects of protein dynamics, including activity, stability, subcellular localization and interactions with partners. SUMO proteins, approximately 10 KDa in size, share a secondary structure closely resembling ubiquitin (Elrouby, 2015). The SUMOylation process parallels ubiquitination, relying on E1, E2 and E3 SUMOylation enzymes to initiate a cascade of reactions. Activation of SUMO proteins by E1 enzymes in an ATP‐dependent manner precedes cleavage of the C‐terminus by SUMO proteases (UPLs), exposing a di‐Gly motif (Chang and Yeh, 2020; Yates *et al*., 2016). In most instances, SUMO forms a connection with the target protein through the Lys in the ѱKxD/E motif or a SUMO‐interacting motif (SIM). The SIM, a short sequence composed of 3–4 hydrophobic residues with an adjacent acidic region (Ser, Thr, Glu, Asp), is commonly present in various proteins (Yau *et al*., 2021). Transcription factors (TFs) are the frequently detected targets of SUMOylation. TF SUMOyaltion is associated with reduced target gene expression due to interference with other post‐translational modifications (PTM) or trigger degradation.

The BES1/BZR1 are the major transcription factor of brassinosteroids (BR) signalling, which control over a diverse array of growth and developmental events (Nolan *et al*., 2020). BES1 and BZR1 feature a basic/helix–loop–helix domain in the N terminal and a PEST domain in the middle portion of the protein (Yin *et al*., 2005). In Arabidopsis, BES1 binds to the E‐box element and BRRE sequence in the promoter region of approximately 1609 target genes, regulating cellular activities and biological processes, including hypocotyl elongation, photomorphogenesis, skotomorphogenesis, xylem development and more (Huang *et al*., 2022; Shi *et al*., 2022). In the context of cotton, GhBES1, GhBES1.4, GhBZR1 and GhBZR3 have been identified as key contributors to the pivotal process of cotton fibre development, particularly during the elongation stage. Additionally, GhBES1.4 has been demonstrated to intricately regulate the expression of a substantial number of genes, encompassing 1531 genes at the genome‐wide level. In Arabidopsis, BES1 targets BR biosynthesis genes *DWARF4* (*DWF4*) and *CPD*, encoding cytochrome P450 enzymes, forming a feedback inhibition loop to modulate BR responses (Yu *et al*., 2011). Other genes activated by BR, such as *ACS5* and *PER5*, are direct targets of BES1, promoting hypocotyl elongation (Wang *et al*., 2018b). Importantly, BES1 has been reported to interact with BOP1 in Arabidopsis, the researchers identified BOP1 as a negative regulator of BR signalling in Brz treatments, BOP1 and BES1 forming a large protein complex in cytosol that impedes BES1 transport from the cytosol to the nucleus (Shimada *et al*., 2015). Therefore, it is intriguing to explore whether this interaction complex is associated with post‐translational modification. Existing research has documented that BES1/BZR1 undergo post‐translational modifications, including SUMOylation, phosphorylation and ubiquitination, to regulate their activity, abundance and nuclear localization (Kono and Yin, 2020; Yang *et al*., 2017). The SUMO E3 ligase SIZ1 mediates the SUMOylation of BES1, regulating its activity and nuclear localization. The SUMOylation site of BES1 is identified at site of 302 lysine. BES1 protein in wild type is less stable than in *siz1‐2* seedlings with or without BR, suggesting the SIZ1‐mediated SUMOylation promotes BES1 instability (Zhang *et al*., 2019a). Contrary to BES1, a new study reported that SUMOylation of BZR1 inhibits its interaction with BIN2 kinase. The BIN2 kinase leading to phosphorylation, instability and degradation of BZR1. SUMO proteases, such as ULP1a, and deSUMOylate BZR1, inhibit plant growth (Srivastava *et al*., 2020). Furthermore, research indicates that ubiquitin E3 ligases SINATs interact with dephosphorylated BES1, mediating its ubiquitination and subsequent degradation (Yang *et al*., 2017). Based on these findings, we hypothesize that BOP1 may play a novel role in BES1 SUMOylation through the BOP1‐BES1 interacting complex, thereby regulating plant growth and development. This potential role of BOP1 in the SUMOylation process could be of greater significance for BES1 degradation compared with the SIZ1‐mediated process.

This study delves into the *BOP* genes in upland cotton, specifically focusing on *GhBOP1*. The findings revealed that overexpression of *GhBOP1* leads to a reduction in fibre length and a semi‐dwarfism phenotype in cotton. Conversely, suppressing *GhBOP1* expression showed a contrary phenotype. GhBOP1 is shown to interact with GhBES1, facilitating its SUMOylation process in an E3 ligase‐like manner and exerting a negative regulatory influence on the BR signalling pathway. By influencing the expression of BR response genes, *GhBOP1* plants effectively modulate both fibre length and plant height in a *GhBES1‐*dependent manner.

## Result

### Structural analysis of the GhBOP1 protein

We isolated the *GhBOP1* gene from upland cotton cDNA, anticipating potential function redundancy with GhBOP2, akin to Arabidopsis (Norberg *et al*., 2005). Sequence homology analysis between cotton BOP and Arabidopsis BOP proteins (Figure S1a) indicated that GhBOP1 exhibits the highest similarity to AtBOP1, sharing a protein sequence identity of 74%, leading to its designation as GhBOP1. As the allotetraploid crop, BOP1 in cotton has two copies, Gh_A09G1115 and Gh_D09G1120 which located in At and Dt subgenomes, respectively. They share 99.4% and 99.6% similarities in coding sequences and amino acid sequences (Zhang *et al*., 2019b). We think Gh_A09G1115 and Gh_D09G1120 are functional redundant in cotton plant architecture, so this research chooses the Gh_A09G1115 as the representation of GhBOP1, the diversity between them need to be futher exploration. Domain analysis revealed that the *BOP* gene encodes two conserved domains, a BTB/POZ (Broad Complex, Tramtrack, and Bric‐a‐brac/POX virus and Zinc finger) domain and four ankyrin motifs (Figure S1a). NPR1 was the first identified protein in BTB/POZ ankyrin family, which function in the salicylic acid induced systemic acquired resistance. NPR3 and NPR4 are paralogs that act primarily to modulate NPR1 abundance for appropriate resist pathogens. BOPs were identified through isolation of a *bop1‐1* mutant with leafy petioles, which further demonstrated involved in leaf and flarol development, comprise a separate subclade with NPR family in the phylogenetic tree.

### Overexpression of *GhBOP1* caused shorter plant architecture in transgenic plants

To explore the physiological role of *GhBOP1*, we initiated overexpression of the intact *GhBOP1* form (Gh_A09G1115.1) in Arabidopsis, resulting in transgenic lines (OE‐1, OE‐2 and OE‐3), designated as GhBOP1‐OX (Figure S2a). Western blot analysis confirmed elevated GhBOP1 expression (Figure S2b), and we examined the phenotypes of GhBOP1‐OX plants (Figure S3a). GhBOP1‐OX plants exhibited a semi‐dwarf plant architecture (Figure S3b,c), featuring shorter rosette petioles and an increased ratio of leaf length to width (Figure S3d,e), resembling the phenotype of AtBOP1‐overexpressed plant (Khan *et al*., 2014). Notably, GhBOP1‐OX‐overexpressed plants were shorter with a lower leaf length/width ratio compared to Col‐0 (Figure S3e). Intriguingly, these phenotypes mirrored those of BR biosynthesis and signalling‐defective mutants like *det2*, *bak1 and bin2*. (Li *et al*., 2001, 2002; Noguchi *et al*., 1999). BRs play a pivotal role in regulating plant growth processes, including cell division, hypocotyl growth, xylem development and photomorphogenesis (Kim and Russinova, 2020; Nolan *et al*., 2020; Planas‐Riverola *et al*., 2019). Consequently, we hypothesized that BR signalling might be inhibited in GhBOP1‐OX plants, so we tested the expression of a set of BR responsive genes by qRT‐PCR. In GhBOP1‐OX plant, the expression of CPD4 and DWF4 was increased while ACS5 and PER5 was reduced compared with that of the wild‐type plant (Figure S4).

**Figure 1:**
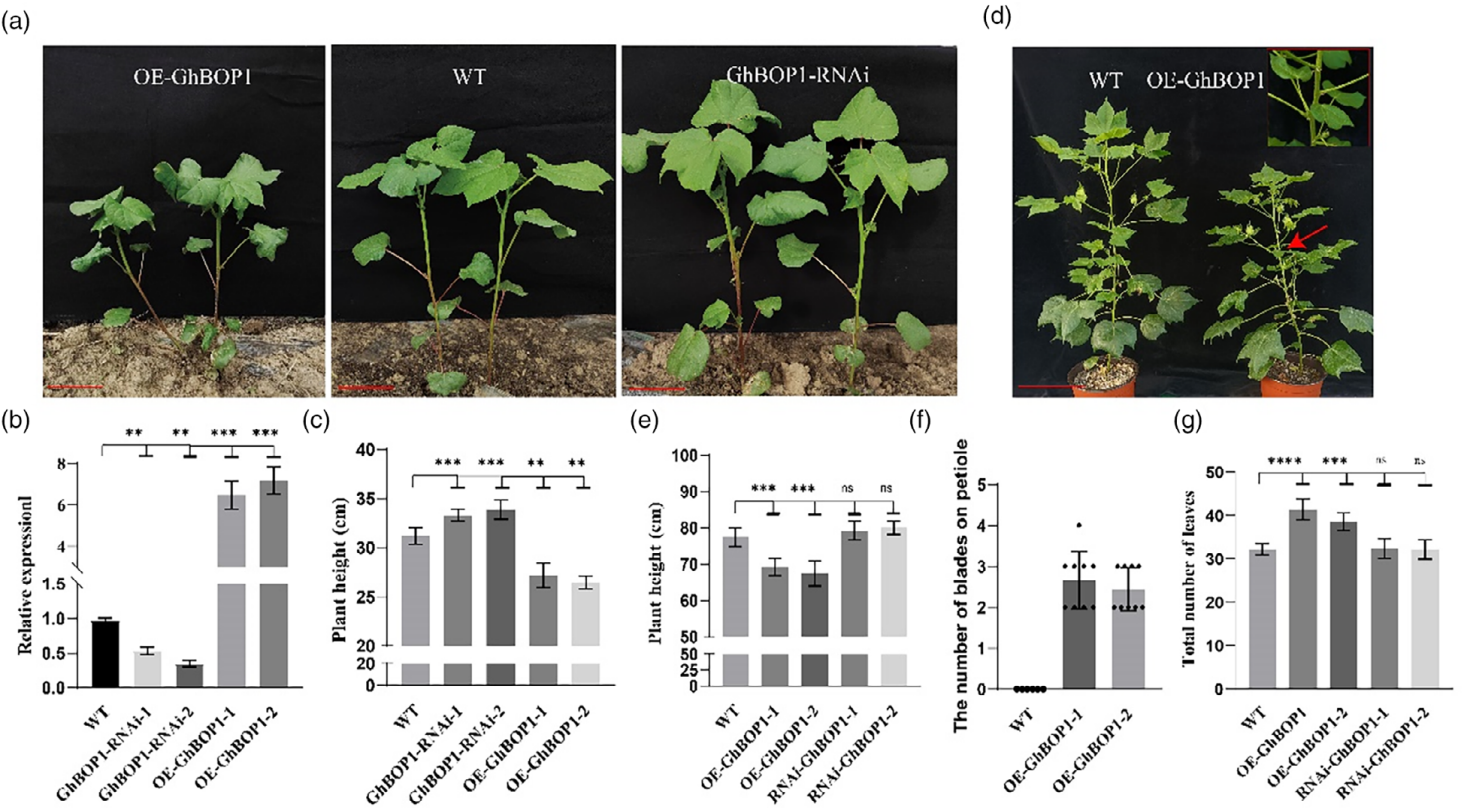
Phenotype analysis of GhBOP1 transgenic upland cotton. (a) Representative image illustrating the plant architecture of 30‐day‐old T2‐generation GhBOP1‐RNAi, OE‐GhBOP1 and WT plants at the vegetable stage under control growth conditions. (b) Quantitative RT‐PCR analysis of the transcription expression level of GhBOP1 in WT, OE‐GhBOP1 and GhBOP1‐RNAi cotton. Data represent the mean ± SD from three independent experiments. GhUb7 was used as the internal control. Students' *t*‐test, *P* < 0.05, three biological replicates. (c), Plant height of (a). The date shown are the means and standard deviation of seven plants (*n* = 7 plant/line). Students' *t*‐test, *P* < 0.05. (d) Representative image illustrating the plant architecture of 60‐day‐old T2‐generation OE‐GhBOP1 and WT plants at the squaring period. (e) Plant height of (d). (f) The number of blades derived from stipules. (g) Total number of leaves of cotton. Data shown are means and standard deviation of five plants (*n* = 5 plant/line). Statistically significant differences between each transgenic line and WT are marked with asterisk(s). ****P < 0.0001, ***P < 0.001, **P < 0.01, *P < 0.05, ns *P* > 0.05.

To assess whether the observed OE‐GhBOP1 phenotypes extended cotton, we placed the *GhBOP1* coding sequence under the control of the cauliflower mosaic virus CaMV 35S promotor and introduced the construct into cotton to generate stable OE‐GhBOP1 transgenic lines. Additionally, we developed RNAi transgenic plants to downregulate *GhBOP1* expression in upland cotton (Figure 1a). Expression analysis confirmed increased *GhBOP1* levels in OE‐GhBOP1 lines and decreased levels in GhBOP1‐RNAi lines (Figure 1b). As anticipated, GhBOP1‐RNAi plants exhibited slightly greater height than WT plants, while OE‐GhBOP1 displayed a shorter plant height phenotype (Figure 1c,d). A detailed phenotypic examination of OE‐GhBOP1 plants revealed more leaflets on the lateral organ boundaries (LOB) at the bud stage, suggesting differentiation of petiole cells of LOB origin (Figure 1e,f). This increase in LOB leaflets contributed to a higher total leaf count (Figure 1g).

### GhBOP1 regulates fibre development

**Figure 2:**
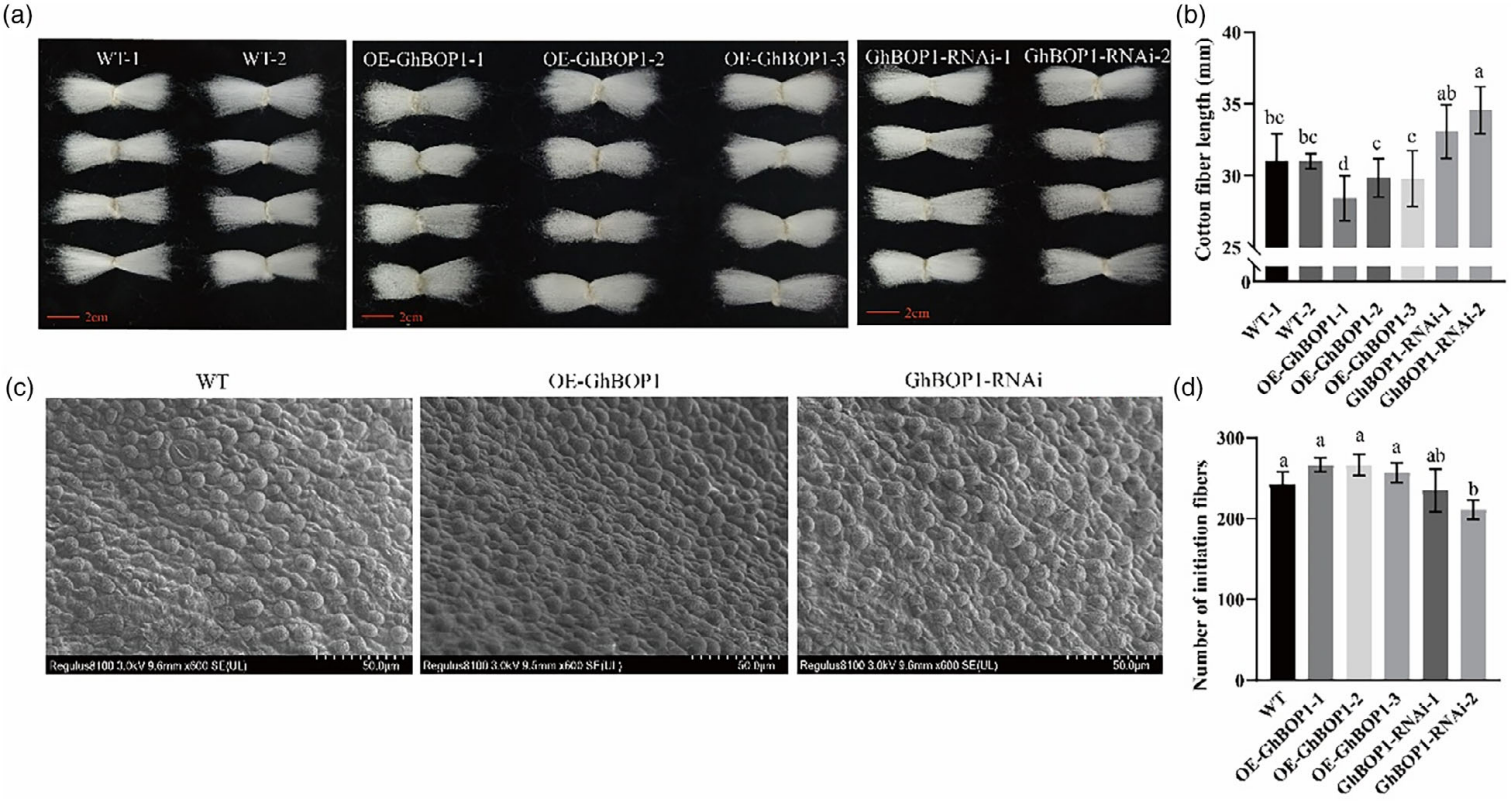
Bolls size and weight analysis of transgenic cotton overexpressing GhBOP1. (a) Phenotype of cotton fibres in the wild type and OE‐GhBOP1 and GhBOP1‐RNAi lines. Scale bar: 1.5 cm. (b) Statistical analysis of the fibre length in the wild type and OE‐GhBOP1 and GhBOP1‐RNAi lines. Differences among normally distributed values of the six experimental groups were analysed by using one‐way ANOVA, followed by Tukey multiple comparisons post‐test. (c) Phenotypes of initiating fibre cells of cotton ovules at 1 day after flowering from the wild type and OE‐GhBOP1 and GhBOP1‐RNAi under 600× scanning electron microscopy. Scale bar: 50 μm. (d) Statistical analysis of the number of initiating fibre cells from the wild type and OE‐GhBOP1 and GhBOP1‐RNAi under 400× scanning electron microscopy. Differences among normally distributed values of the six experimental groups were analysed by using one‐way ANOVA, followed by Tukey multiple comparisons post‐test.

Cotton fibre, elongated trichomes originating from the ovule epidermis, plays a pivotal role in the textile industry. To unravel the role of *GhBOP1* in fibre development, we assessed fibre length in transgenic cotton lines (Figure 2a). Fibres from OE‐GhBOP1 plants exhibited an average length of 28.4 ± 1.6 mm, which was 2 mm shorter than control plants and 4.5 mm shorter than GhBOP1‐RNAi lines (Figure 2b). To observe fibre initiation cells more clearly, we dissected ovules on the day of cotton flowering, fixing them for scanning electron microscopy. As shown in Figure 2c, OE‐GhBOP1 ovules displayed slightly more extensive fibre protuberance than those in WT and GhBOP1‐RNAi lines. Quantitative analysis confirmed that OE‐GhBOP1 exhibited a slightly higher count of initiation fibre cells compared to other transgenic lines (Figure 2d). Moreover, we measured the yield traits and fibre quality traits of transgenic cotton, the length of upper quartile fibre in OE‐GhBOP1 lines was also shorter over that of ZM35 fibres, the uniformity index, micronaire and lint percentage were no significant difference among them (Table S1). Previous studies have reported the involvement of the BR transcriptional regulatory network in regulating fibre elongation in cotton (Liu *et al*., 2022; Yang *et al*., 2023). We measured the expression level of GhAP2L, GhbHLH282 and GhEXP8 in OE‐GhBOP1, WT and GhBOP1‐RNAi lines, those genes were reported promoted cotton fibre elongation while involved in BR signalling (Liu *et al*., 2020; Lu *et al*., 2022; Wang *et al*., 2020b; Wen *et al*., 2023). We found that the expression of GhAP2L, GhbHLH282 and GhEXP8 were increased in GhBOP1‐RNAi lines (Figure S5). Collectively, these findings establish *GhBOP1* as a regulator of cotton fibre initiation and elongation, with implications for the BR signal pathway.

### GhBOP1 negatively regulates BR signalling in cotton

**Figure 3:**
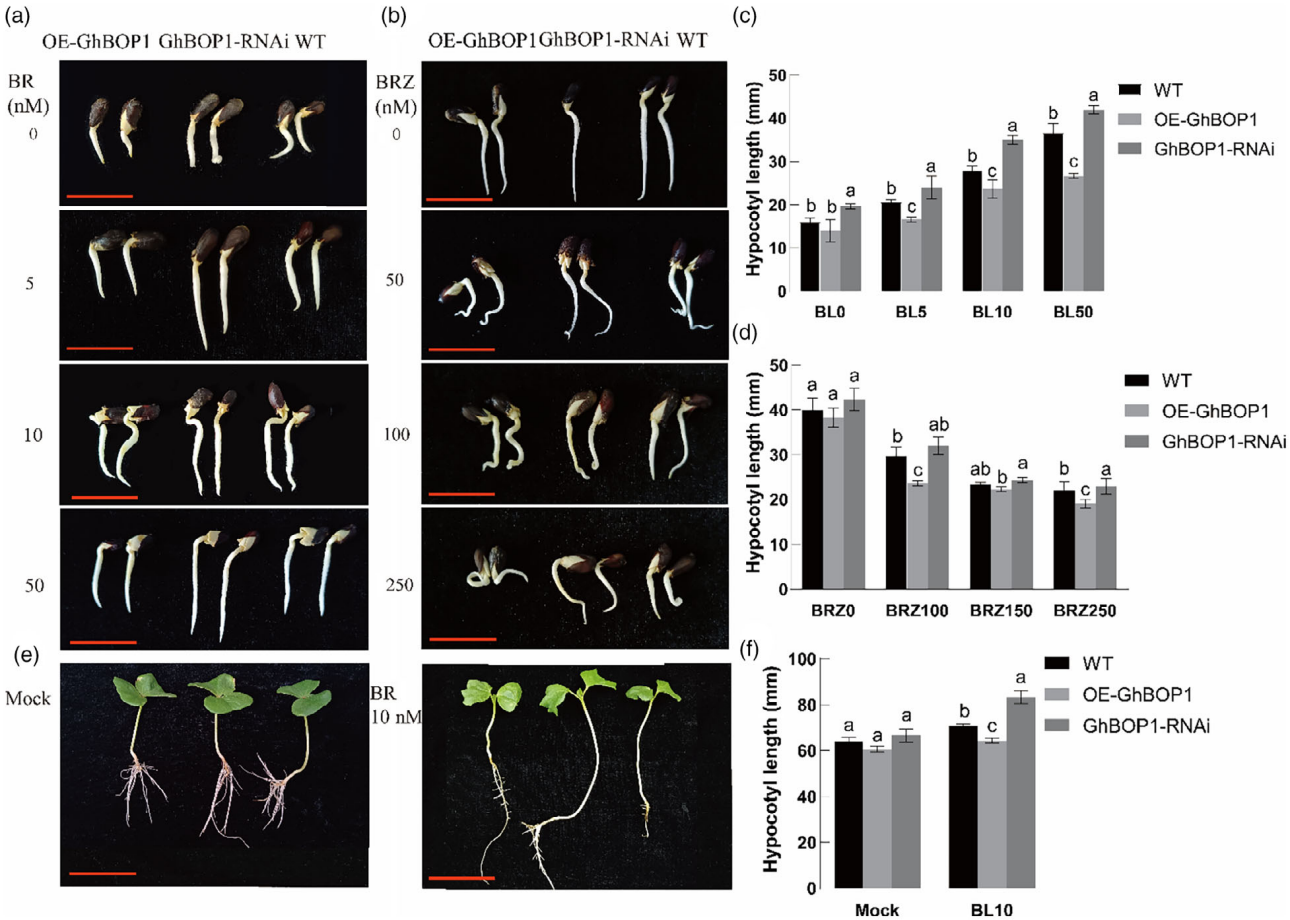
GhBOP1 negatively regulates BR signalling in upland cotton. (a) representative picture of the phenotype of 2‐day‐old light‐grown WT, OE‐GhBOP1 and GhBOP1‐RNAi seedings in the presence of different concentrations of BL. Scale bar, 2.0 cm. (b) representative picture of the phenotype of 3‐day‐old dark‐grown WT, OE‐GhBOP1 and GhBOP1‐RNAi seedings in the presence of different concentrations of BRZ. Scale bar, 4.0 cm. (c) the hypocotyl lengths of 2‐day‐old light‐grown seedings in the presence of different concentrations of BL. Data were the means of 10 seedings. Significant differences were based on the student's *t*‐test (*P < 0.05, **P < 0.01). (d) The hypocotyl lengths of 3‐day‐old light‐grown seedings in the presence of different concentrations of BL. Data were the means of 10 seedings. (e) The presentative picture of the phenotype of 7‐day‐old light‐grown WT, OE‐GhBOP1 and GhBOP1‐RNAi seedings in the presence of different concentrations of BL. Scale bar, 6.0 cm. (f) The hypocotyl lengths of 7‐day‐old light‐grown seedings in the presence of different concentrations of BL. Data were the means of 10 seedlings.

To elucidate the involvement of *GhBOP1* in the BR signalling pathway and its role in BR responses, cotton seedlings from OE‐GhBOP1, WT and GhBOP1‐RNAi lines were subjected to treatments with varying concentrations of BL or brassinazole (BRZ). BL, the most active BR form purified from rapeseed pollen (Nolan *et al*., 2020), was sprayed on seedlings after germination. Notably, GhBOP1‐RNAi seedlings exhibited longer hypocotyls than those in WT and OE‐GhBOP1, indicating increased sensitivity to BL in GhBOP1‐RNAi plants (Figure 3a). Conversely, when seedlings were treated with different concentrations of BRZ, a specific BR biosynthesis inhibitor (Asami *et al*., 2000), and incubated in the dark for 12 h, OE‐GhBOP1 seedlings displayed heightened sensitivity to BRZ compared with WT, exhibiting a hook phenotype due to dark treatment (Figure 3b). Statistical analysis revealed that the hypocotyl length in the BL50 (50 nM) and BRZ100 (100 nM) treatment groups exhibited more significant changes than those in other concentration treatment groups (Figure 3c,d). To further confirm the efficacy of BL, radicles from 5‐day‐old cotton seedlings (Figure 3e leaf) were treated with 10 nM BR under 10 h of darkness, and then transferred to plastic pots for a 2‐day water culture. Radicle length in GhBOP1‐RNAi was 20 mm longer than that in WT, while OE‐GhBOP1 plants displayed shorter radicles (Figure 3e,f), supporting the earlier findings. In addition, we analysed the expression of BR‐responsive genes GhCPD and GhACS5 when the seedlings treated with BL50 or BRZ100. The BR‐repressed GhCPD was downregulated while the BR‐induced GhACS5 in the GhBOP1‐RNA line compared with the wild‐type (Figure S6). These results collectively indicate that *GhBOP1* exerts a negative regulatory role in the BR signalling pathway.

### GhBOP1 is a SUMO‐binding protein

**Figure 4:**
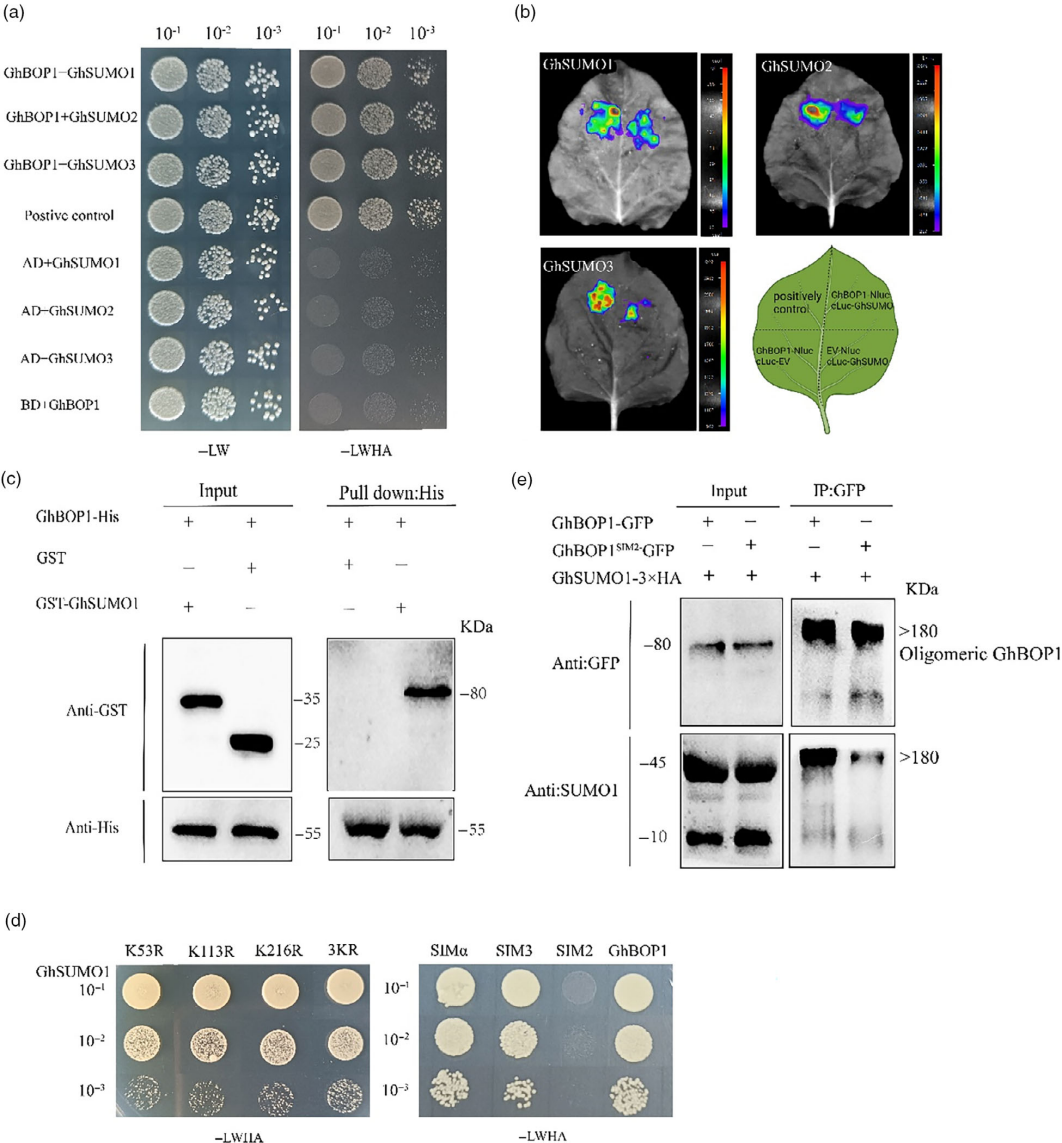
GhBOP1 interacts with GhSUMO1. (a) Yeast two‐hybrid. The same concentration of AH109 yeast cells carrying BD‐GhBOP1 and AD‐GhSUMO proteins (GhSUMO1, GhSUMO2 or GhSUMO3) were spotted on SD‐LW and SD‐LWHA. The photographs were taken after 3 days at 28 °C. (b) The luciferase complementary assays. The GhBOP1 and GhSUMO proteins (GhSUMO1, GhSUMO2 or GhSUMO3) were fused to either the C‐ or N‐terminal half of luciferase (cLuc or nLuc), and transiently expressed in *N. benthamiana*. The luminescence signal was detected by applying 1 mM luciferin after 60 h of agroinfiltration. The positive control was constructed according to Chen *et al*. (2008). The coloured scale indicated the intensity of luciferin activity. (c) Pull‐down assay. GhBOP1‐HIS was used to pull down interacting proteins. GST or GST‐GhSUMO1 fusion proteins were detected by Western blotting with anti‐GST antibodies. Asterisks indicate GST‐GhSUMO1 interacted with GhBOP1‐HIS. (d) The different concentrations of AH109 yeast cells that carried BD‐GhSUMO1 and AD‐GhBOP1 and its mutations (K53R, K113R and K216R, simα, sim3 and sim2) were spotted on the SD‐LWHA medium. (e) Co‐IP from *N. benthamiana* leaves expressing fusion proteins GhSUMO1 and GhBOP1‐GFP or GhBOP1^sim2^‐GFP. Proteins were purified with agarose GFP beads and analysed by Western blot using α‐SUMO1 and α‐GFP antibodies.

As *GhBOP1* encodes a BTB/POZ ankyrin repeat protein known to function as an E3 ubiquitin ligase with CUL3 (Wang *et al*., 2020c), we hypothesized its involvement in post‐translational modification processes. Given the reported interaction of BOP1 with BIL/BZR1 or its homologue, BES1, forming a large protein complex in Arabidopsis, we speculated a similar interaction between GhBOP1 and GhBES1 in cotton. Y2H and LCI analyses initially confirmed the interaction between GhBOP1 and GhBES1 (Figure S7). In upland cotton, we initially assessed the interaction between GhBOP1 and GhSUMOs using Y2H. Given that *G. hirsutum* encodes three functional SUMO proteins, GhBOP1 demonstrated interaction with all three (Figure 4a). To verify GhBOP1‐GhSUMOs interaction in plants, a luciferase complementary assay was performed by co‐expressing GhBOP1 with GhSUMO1, GhSUMO2 or GhSUMO3 fusion proteins in *N. benthamiana* leaves. Notably, GhSUMO1 exhibited a stronger interaction with GhBOP1 compared with other SUMO proteins (Figure 4b). Subsequently, a pull‐down assay using recombinant GST‐GhSUMO1 and GhBOP1‐HIS purified from *Escherichia coli* demonstrated that GST‐GhSUMO1 could be pulled down by GhBOP1‐HIS (Figure 4c).

To elucidate the interaction mechanism between GhBOP1 and GhSUMO1, we predicted three lysines potentially targeted for GhSUMO1 conjugation using JASSA software (Figure S8). Mutating each lysine (K) to arginine (R) or in combinations did not block or reduce the interaction in Y2H (Figure 4d), suggesting that GhBOP1 may interact with GhSUMO1 through a SIM sequence. GhBOP1 harboured three putative SIM sequences, conserved in planta (Figure S9). Mutating all hydrophobic residues to alanine (A), revealed that only the sim2 mutant blocked the GhBOP1‐GhSUMO1 interaction in yeast cells (Figure 4d), indicating that SIM2 is likely the sole functional motif interacting with GhSUMO1.

To validate the GhBOP1‐GhSUMO1 interaction through SIM2 in plants, we performed a Co‐IP assay by co‐expressing GhBOP1 or GhBOP1^sim2^ and GhSUMO1 fusion proteins in *N. benthamiana*. After purifying GFP‐tagged GhBOP1 and GhBOP1^sim2^ fragments, we observed that GhBOP1‐GFP preferentially retained a higher abundance of GhSUMO1 (Figure 4e). In summary, our data demonstrate that GhBOP1 binds to GhSUMO1 in a non‐covalent manner, dependent on the SIM2 motif of GhBOP1.

### GhBOP1 mediates the SUMOylation of GhBES1

**Figure 5:**
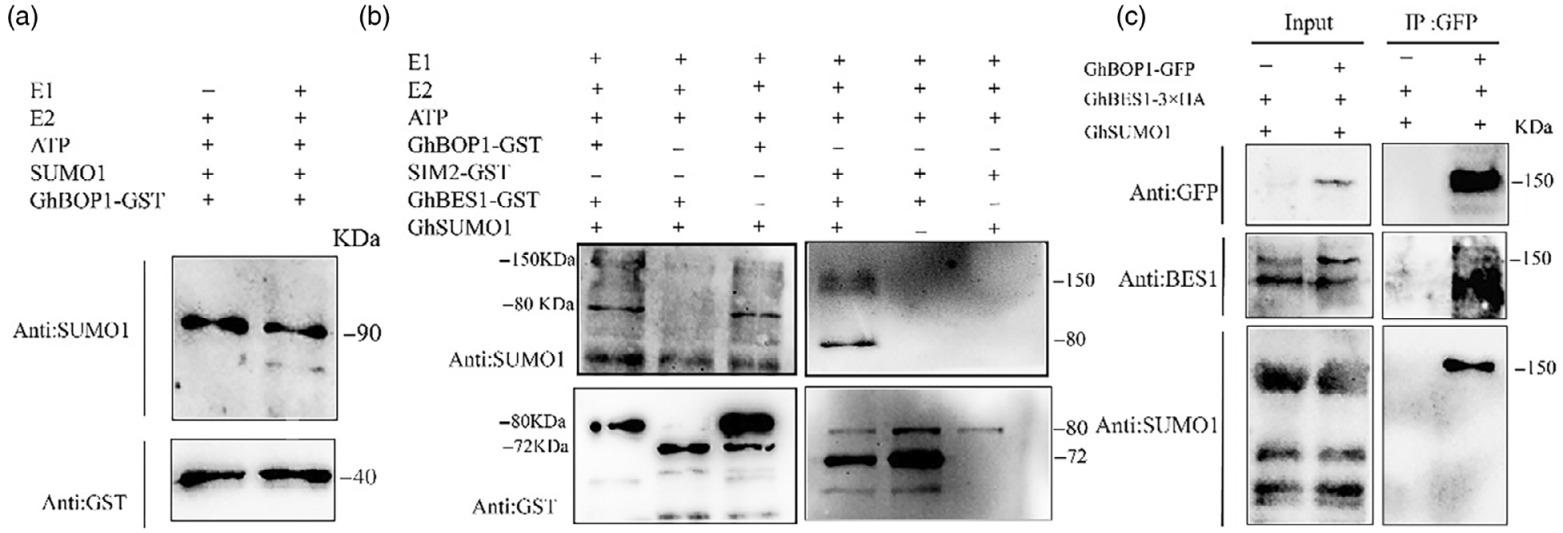
GhBOP1 mediates the SUMOylation of GhBES1 *in vitro* and *in vivo*. (a) and (b), SUMOylation assay *in vitro*. SUMO E1 activation enzyme, SUMO E2 conjugation enzyme, SUMO proteins and ATP were contents from the SUMOylation kit. Fusion proteins GhBOP1‐GST and GhBES1‐GST were extracted from *E. coli* BL21 cells. Proteins were purified using agarose GST beads and analysed by Western blot using α‐GST and α‐SUMO1 antibodies. (c) SUMOylation assay *in vivo*. Fusion proteins GhSUMO1, GhBOP1‐GFP and GhBES1 were co‐expressing in *Nicotiana bethamiana* leaves. Samples were adjusted for equal amounts of total protein and IP samples were analysed by Western blot using α‐SUMO1 and α‐GFP antibodies.

We initially investigated the potential SUMOylation of GhBOP1, considering its similarity to other SIM‐containing proteins with reported SUMOylation, and its interaction with SUMOylated proteins. In an *in vitro* SUMOylation reconstitution assay using SUMOylation Kit, we incubated purified GhBOP1‐His with E1 (SAE1b and SAE2), E2 (UBC9), SUMO1 and ATP. However, no multiple SUMOylated GhBOP1 bands or any migrated forms were detected (Figure 5a), indicating that GhBOP1 itself cannot undergo SUMOylation. Intriguingly, when GhBES1 was introduced into the reaction system, specific SUMOylation of GhBES1 occurred within the GhBES1‐GhBOP1‐GhSUMO1 complex. Notably, GhBES1 SUMOylation was not observed in the absence of GhBOP1 (Figure 5b). This result suggests that GhBES1 SUMOylation *in vitro* relies on the presence of GhBOP1.

We further explored GhBES1 SUMOylation *in planta* by co‐expressing GhBOP1‐GFP, GhBES1‐HA and GhSUMO1‐HA fusion proteins in *N. benthamiana* leaves. Following enrichment with GFP beads, SUMOylation of GhBES1 was detected using anti‐BES1 antibodies (Figure 5c). Our findings indicate that GhBES1SUMOylation is dependent on GhBOP1. The specific interaction observed among GhBOP1, SUMO and GhBES1 suggests that GhBOP1 functions akin to an E3 ligase‐like protein.

### GhBOP1 promotes the degradation of GhBES1

**Figure 6:**
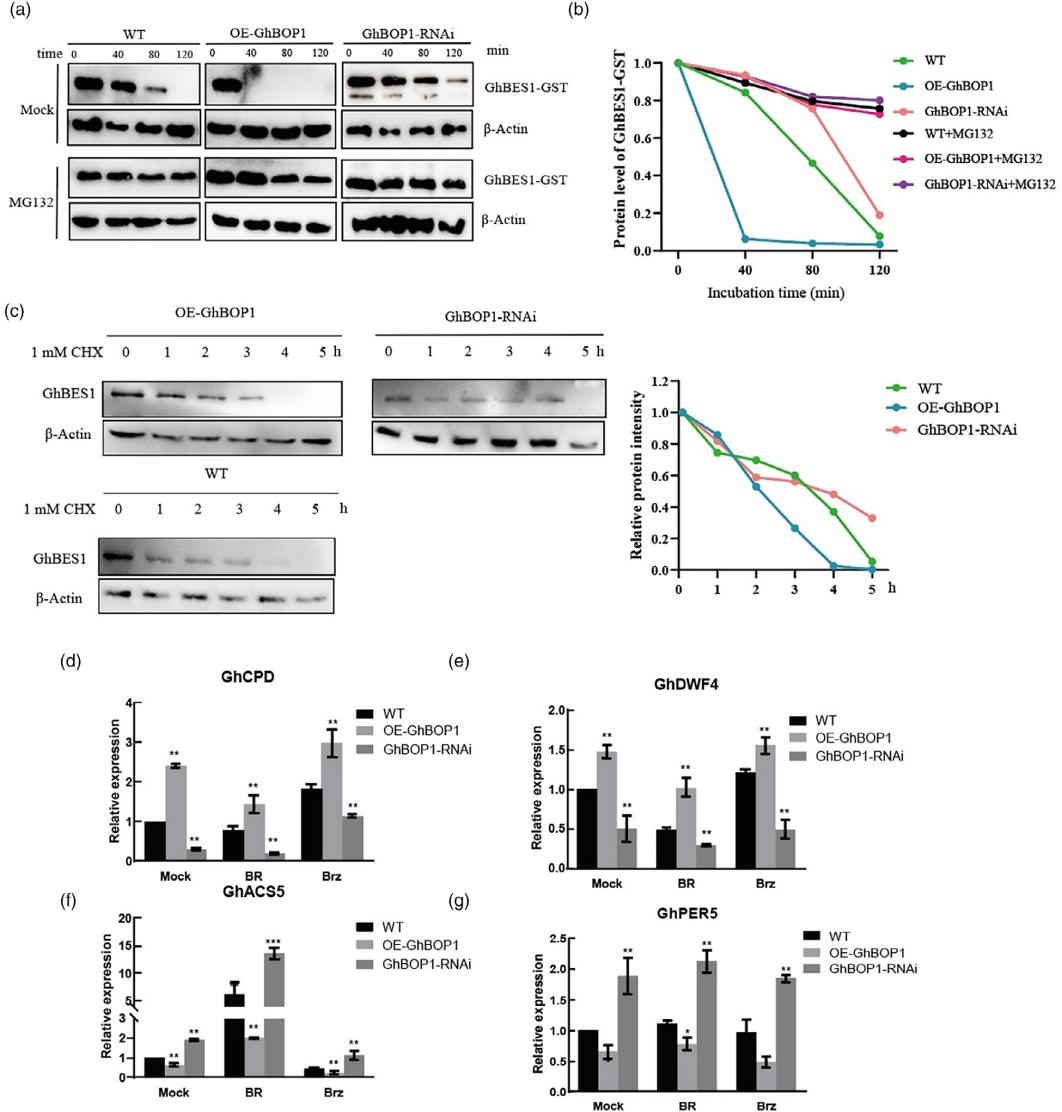
GhBES1 Promoted the degradation of GhBES1. (a) Cell‐free degradation assay showed the degradation rate of GhBES1‐GST in WT and transgenic seedlings (OE‐GhBOP1 or GhBOP1‐RNAi), and MG132 inhibited the degradation of GhBES1‐GST in all reactions. GhBES1‐GST protein was detected by agarose GST antibodies. β‐Actin was used for equal loading. (b) Using Image J software (https://imagej.nih.gov/ij/). GhBES1‐GST protein levels at 0 min were defined as ‘1.0’. (c) GhBES1 levels in WT, OE‐GhBOP1 and GhBOP1‐RNAi lines, the 10‐day‐old seedlings were sprayed with 50 μg/mL CHX for the indicated time. (d) BRZ and BL responsiveness of GhDWF4 in WT, OE‐GhBOP1 and GhBOP1‐RNAi. The expression level of GhDWF4 was analysed by qRT‐PCR in 7‐day‐old seedings and treated with DMSO (Mock), 1 μM BRZ or 100 nM BL for 3 h. The data represents means for three independent replications. (e) The expression level of GhCPD in WT, OE‐GhBOP1 and GhBOP1‐RNAi seedings after being treated with DMSO, 1 μM BRZ or 100 nM‐BL for 3 h. (f) The expression level of GhACS5 in WT, OE‐GhBOP1 and GhBOP1‐RNAi seedings after being treated with DMSO, 1 μM BRZ or 100 nM‐BL for 3 h. (g) The expression level of GhEXP8 in WT, OE‐GhBOP1 and GhBOP1‐RNAi seedings after being treated with DMSO, 1 μM BRZ or 100 nM‐BL for 3 h. All data of (d, e) were analysed using Students' *t*‐test with GraphPad Prism, ****P < 0.0001, ***P < 0.001, **P < 0.01, *P < 0.05, ns *P* > 0.05.

SUMOylation is known to exert significant biological effects, influencing protein activity, localization, stability and blocking protein–protein interactions (Müller *et al*., 2001; Novatchkova *et al*., 2004; Wang *et al*., 2020a). To investigate the impact of SUMOylation on GhBES1 stability, a cell‐free degradation assay was conducted. Recombinant GhBES1 protein was incubated in total protein extracts from 10‐day‐old WT or transgenic (OE‐GhBOP1 and GhBOP1‐RNAi) cotton seedlings. A negative control group treated with MG132, an inhibitor of the 26S proteasome complex, was included. Without MG132 treatment, the GhBES1‐GST recombination protein exhibited a faster degradation rate in OE‐GhBOP1 seedlings compared to other seedlings after the indicated time (Figure 6a). Data revealed complete degradation of GhBES1 abundance in the total protein extracted from OE‐GhBOP1 plants within 40 min (Figure 6b). When MG132 was added to the reaction, GhBES1‐GST degradation was inhibited in all seedlings, suggesting GhBOP1‐mediated GhBES1 degradation in a proteasome‐dependent manner. Parallelly, seedlings were treated with the protein synthesis inhibitor cycloheximide for a degradation ratio assessment, revealing higher GhBES1 protein accumulation in GhBOP1‐RNAi plants than in WT seedlings over time (Figure 6a). In OE‐GhBOP1 seedlings, GhBES1 protein levels were lower than those in WT seedlings, affirming that GhBOP1 facilitates GhBES1 degradation.

To further support these findings, the expression of BR‐responsive genes was analysed via qRT‐PCR. In Arabidopsis, BR biosynthesis genes *CPD* and *DWARF4* (*DWF4*) are target genes of BES1, forming a negative feedback loop where BES1 binds to their promoter region and restrains their expression when endogenous BR reaches a certain level (Shigeta *et al*., 2014). Homologous genes were identified in upland cotton (Liu *et al*., 2021), In OE‐GhBOP1 plants, the expression levels of the BR‐repressed genes *GhCPD* and *GhDWF4* were upregulated (Figure 6d,e), while the expression levels of the BR‐inducible genes *GhACS5 and GhPER5* were downregulated compared to the WT and GhBOP1‐RNAi lines (Figure 6f,g). In GhBOP1‐RNAi plants, the expression of *GhDWF4* and *GhCPD* was reduced. As BR‐responsive genes, the expression of *GhACS5* and *GhPER5* was increased by treatment with BL and suppressed by treatment with BRZ in WT plants. These results provide evidence that GhBOP1 promotes the SUMOylation of GhBES1, influencing the transcription levels of *GhBES1* target genes.

### *GhBOP1* epistatically regulates GhBES1 to regulate plant growth

**Figure 7:**
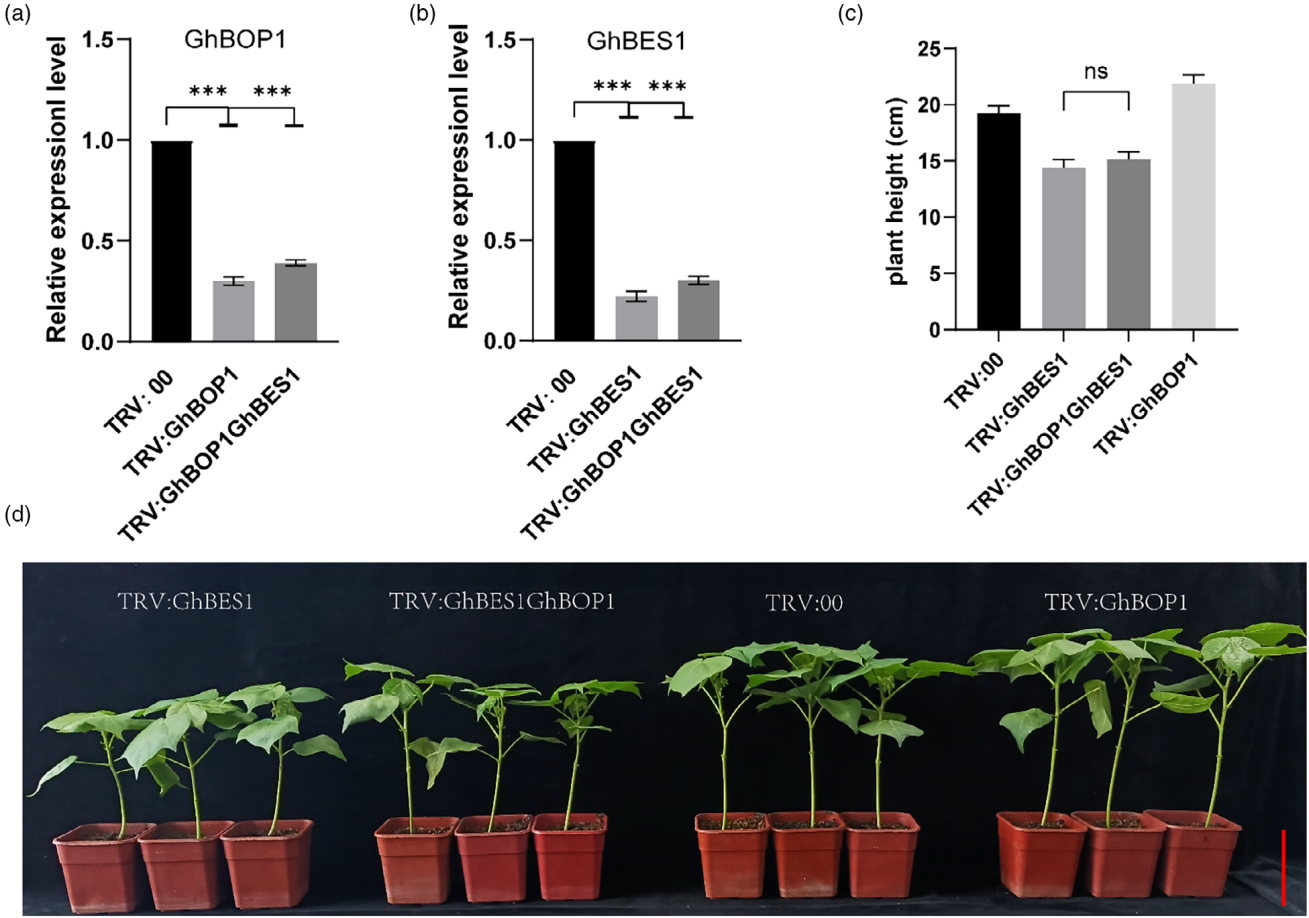
GhBOP1 functions through GhBES1 to regulate plant growth. (a) Relative expression levels of GhBOP1 in the TRV:00, TRV: GhBOP1, TRV: GhBOP1GhBES1 lines. (b) Relative expression levels of GhBES1 in the TRV:00, TRV: GhBES1, TRV: GhBOP1GhBES1 plants. Error bars indicate the standard error of the independent biological replicates. *P < 0.05, **P < 0.01. (c) Statistical analysis of the plant height in TRV:00, TRV: GhBES1, TRV: GhBOP1, TRV: GhBOP1GhBES1 plants. More than 35 plants were used for statistical analysis. (d) The plant height of 21‐day‐old cotton in TRV:00, TRV: GhBES1, TRV: GhBOP1 and TRV: GhBOP1GhBES1 plants. Scale bar, 5 cm.

Both biochemical and genetic experiments have conclusively established that GhBOP1 interacts with GhBES1, facilitating the SUMOylation of GhBES1 and influencing its stability. To investigate whether the biological functions of GhBOP1 require GhBES1, we silenced *GhBOP1* and *GhBES1* genes using a virus (Tobacco rattle virus) induced gene silencing strategy. qRT‐PCR validation confirmed a significant reduction in the expression levels of both *GhBOP1* and *GhBES1* in TRV: *GhBOP1GhBES1* plants, comparable to the levels observed in TRV: *GhBOP1* or TRV: *GhBES1* plants (Figure 7a,b). Phenotypic analysis of 2‐week‐old cotton plants showed that the height of TRV: *GhBOP1* exceeded that of WT plants, mirroring the BR‐enhanced phenotype observed in GhBOP1‐RNAi. Conversely, the plant height of TRV: *GhBES1* was significantly suppressed, consistent with the inhibition of the BR pathway reported by Yang *et al*. (2014). Notably, the plant height of TRV: *GhBOP1GhBES1* plants resembled that of TRV: *GhBES1* but was shorter than TRV: *GhBOP1* plants, suggesting that the knockdown of *GhBOP1* has a mild impact on the function of low GhBES1 (Figure 7c,d). Integrating the biochemical assay data, we can infer that *GhBOP1* is genetically epistatic to *GhBES1*, regulating plant height in a GhBES1‐dependent manner.

## Discussion

We aim to demonstrate that *GhBOP1* functions as a SUMO E3 ligase or, at the very least, as a component of a SUMO E3 ligase complex. In Arabidopsis, only four SUMO E3 ligases have been identified: SIZ1 (SAP AND MIZ1 DOMAIN‐CONTAINING LIGASE), METHYL METHANESULFONATE‐SENSITIVITY PROTEIN 21 (MMS21s) and PROTEIN INHIBITOR OF ACTIVATED STAT‐LIKE (PIALs). They all have the Siz/PIAS RING (SP‐RING) domain which allowed the interaction with E2 enzymes. We found that GhBOP1 have little sequence similarity with them. Interestingly, we observed that GhBES1 could only be SUMOylated under in vitro conditions when incubated with GhBOP1. We speculate both the BTB and SIM domains are required for this SUMOyaltion towards GhBES1. Other BTB proteins have also identified as a SUMO E3 ligase. Its reported that SLX4 complex have SUMO E3 ligases activity and which relies on its SIM and BTB domain (Guervilly *et al*., 2015). In mammalian cells, ZBTB16 (zinc‐finger and BTB domain‐containing protein 16) was also identified promoting the SUMOylation of ACS (apoptosis‐associated speck‐like protein containing a CARD) and regulated assemble the inflammasome (Dong *et al*., 2023). An intriguing aspect of our finding is GhBOP1 absence the SP‐RING domain but also promoting the SUMOylation of GhBES1, may be GhBOP1 is a component of SUMO E3 ligase and facilitating the conjugation of SUMO to their substrate, thereby promoting the SUMOylation process. On the base of direct interaction between GhSUMO1 and GhBOP1 (Figure 4a–c), GhBOP1 may facilitate the formation of a ternary complex involving GhSUMO1 and GhBES1. Subsequently, the SUMOylated GhBES1 is targeted by 26S proteasome and undergoes degradation. A recent study reported an increase in AtBOP1‐AtBES1 puncta in the cytosol, suggesting that large complexes cannot traverse the nuclear pore complex. Building on our findings, we hypothesize that SUMOylated BES1 is also degraded, preventing the complex from being transported into the nucleus. Notable, the specificity interactions like E3‐substrate are crucial for the nonconsensus attachment of SUMOylation (Gareau and Lima, 2010). In our study, mutating the hydrophobic residues in the SIM2 motif blocked the interaction between GhSUMO1 and GhBOP1, while mutating the lysine sites in the GhBOP1 sequence did not affect the interaction (Figure 4e). SIM sequence is present in almost all RING SUMO E3 ligases and plays a pivotal role in regulating enzyme activity (Reverter and Lima, 2005). SIMs anchor the donor SUMO in a favourable orientation to conjunct with the substrate from the E2‐SUMO complex (Plechanovová *et al*., 2012). Our study demonstrated that the SIM2 motif is also crucial for GhBOP1 activity *in vivo* and *in vitro* (Figure 4d,e). Previous reports have indicated that BOP1 mediates the ubiquitination of PIF4 and LEAFY in a CUL3‐based ubiquitin E3 ligase (Chahtane *et al*., 2018; Zhang *et al*., 2017). However, our study is the first to report that BOP1 functions like a SUMO E3 ligase. In Arabidopsis, it has been reported that the SUMOylation of BES1 by SUMO E3 ligase SIZ1 destabilizes and inhibits BES1 activity (Zhang *et al*., 2019a). Notably, SIZ1 as an E3 ligase was not required *in vitro* assays. In our study, we discovered that the SUMOylation of cotton GhBES1 depends on GhBOP1. Therefore, we concluded that GhBOP1 acts as a SUMO E3 ligase targeting its partners, employing a mechanism that requires further elucidation.

The degradation of proteins by the 26S proteasome is a crucial process in hormone signalling, chromatin structure and the morphogenesis of plant architecture (Vierstra, 2009). Transcription factors and their co‐activators often undergo degradation by the 26S proteasome, leading to changes in the expression of downstream target genes (Collins and Tansey, 2006). BES1 contains a PEST domain in its N‐terminal region, suggesting that it is targeted for degradation by 26S proteasome (Rechsteiner and Rogers, 1996). Our study revealed that GhBOP1 mediates the SUMOylation of GhBES1, promoting its degradation (Figure 6a,b). Other studies have reported that various PTMs including phosphorylation and ubiquitination, can also influence the stability of BES1 (Wang *et al*., 2021; Yin *et al*., 2002). We speculated that phosphorylation and SUMOylation may collaborate in regulating protein activity. As BES1 is a key transcription factor in the BR signalling pathway, typically binding to E‐box (CANNTG) or BRRE (CGTGT/CG) elements in promoters (He *et al*., 2005; Yin *et al*., 2005), it regulates the expression of 1609 target genes in the BR transcription network (Yu *et al*., 2011), including BR biosynthesis genes *CPD*, *DWF4*, as well as BR response genes *ACS5* and *PER5*. Changes in the transcription expression of these genes were observed in transgenic cotton plants treated with BL or BRZ (Figure 6c–f). The results suggest that GhBOP1 negatively regulates the BR signalling pathway by promoting the degradation of GhBES1. This study unveils the regulatory role of GhBOP1 in regulating plant architecture, including plant height, and branching pattern and fibre length (Figure 2, Figure S3). As known, BRs play a positive role in modulating plant architecture including leaf and root growth and promoting fibre elongation in cotton (Gudesblat and Russinova, 2011; Liu *et al*., 2022; Sun *et al*., 2005; Yang *et al*., 2023). In Arabidopsis, *AtCPD* and *AtDWF4*, associated with the BR biosynthesis pathway, encode cytochrome P450 superfamily protein (Azpiroz *et al*., 1998; Choe *et al*., 1998). Our findings indicate that the overexpression of *GhBOP1* upregulates the expression of *GhCPD* and *GhDWF4* (Figure 6c,d), suggesting suppression of the BR signal in the transgenic lines. Treatment with BL rescues the expression of these genes. Notably, OE‐GhBOP1 plants displayed reduced plant height in both vegetative and reproduction stages, accompanied by decreased internode length (Figure 2a). To assess the role of GhBOP1‐mediate BR signalling in regulating plant height, we employed the VIGS approach to silence both GhBES1 and GhBOP1 in cotton and analysed the phenotype. The plant height of TRV: *GhBES1GhBOP1* is similar to that in TRV: BES1, indicating that GhBOP1 is genetically epistatic to GhBES1, and the regulation of plant height depends on GhBES1 (Figure 7a,c,d).

In cotton, plant height is one of major factors influencing the yield, and this trait is helpful for mechanized harvest in the main cotton production areas. The plant architecture traits were evaluated when overexpression the *GhBOP1*, which include shorter plant height and more blade‐on‐petioles. And the fibre length was longer when knock down the expression of GhBOP1. Taken together, our study provides evidence that GhBOP1 mediates the repression of BR signalling by promoting the SUMOylation of GhBES1, influencing fibre development and plant architecture in cotton. Molecular studies revealed that GhBOP1 negatively regulates the BR signalling pathway. Further biochemical assays demonstrated that GhBOP1 interacts with GhBES1 and promotes its SUMOylation, leading to the degradation of SUMOylated GhBES1 by the proteasome (Figure S10). The findings reported in this study propose a novel mechanism of GhBES1 SUMOylation and provide a strategy for improving cotton plant architecture and fibre quality.

## Experimental procedures

### Plant materials and growth conditions

The upland cotton (*G. hirsutum*) cultivar ZM 35 (WT) and the transgenic lines *OE‐GhBOP1* and *GhBOP1‐RNAi* regenerated from Zhongmian 35 were cultivated in the greenhouse for 6 months under 16‐h light/8‐h dark photoperiod at 28 °C. Plant architecture characteristics, such as plant height, blade petiole and total number of blades in *OE‐GhBOP1*, were assessed at reproductive and harvest stages, respectively. Cotton plants designated for virus‐induced gene silencing (VIGS) analysis were individually potted under control conditions.

*Arabidopsis thaliana* ecotype Columbia‐0 (Col‐0) served as the wild‐type plant and was grown at 22 °C under a 16‐h light/8‐h dark photoperiod. Transgenic seeds were germinated on half‐strength Murashige and Skoog (1/2 MS) medium after completing the vernalization phase at 4 °C. After 1 week of growth, the seedings were transferred to the soil. Tobacco (*Nicotiana benthamiana*) plants were cultivated under the same conditions as *A. thaliana* before transient expression analysis.

### Plasmid construction

The full‐length coding sequence (CDS) of *Gh_A09G137700/GhBOP1* was PCR‐amplified from the cotton cultivar ZM35, utilizing primers designed through Primer 5.0 based on the sequence from the cotton database CottonFGD (CottonFGD: https://cottonfgd.net/). Subsequently, the CDS was cloned into the binary expression vector pCambia1302. To preserve *GhBOP1* protein expression, a linker sequence (GGGSSGGG) was inserted between *GhBOP1* and the *GFP* sequence. The specific sequence of *GhBOP1* was then incorporated into the RNAi vector pHANNIBAL to generate the GhBOP1‐RNAi vector. For the purification of recombinant protein and pull‐down assays, the coding regions of GhBOP1, GhBES1 and GhSUMO1 were cloned into pET‐28a and pGEX‐6p‐1, respectively. A tobacco rattle virus (TRV)‐induced silencing system was developed to knock down the expression of GhBOP1 and GhBES1 (Liu *et al*., 2002).

### Generating transgenic plants

The genetic transformation of *A. thaliana* was conducted through the floral dip method (Clough and Bent, 1998). In brief, the *pCambia1302‐GhBOP1* recombinant plasmids underwent sequencing verification before being introduced into *Agrobacterium tumefaciens* GV3101. The recombinant strains were utilized to transform WT plants twice within a 5‐day interval. Transgenic lines were selected on 1/2 MS medium supplemented with 50 μg/mL kanamycin. Upon seedling maturation, the plants were transferred to soil, and transformants were identified through qRT‐PCR and Western blot analysis.

### Phenotypic analysis

In transgenic Arabidopsis, the leaf area, the leaf length and leaf width were measured using photographs and analysed by ImageJ software. The rosette leaves of Arabidopsis were three‐leaf whorled, the fifth leaves were the longest and widest leaves among the second wheel leaves. For petiole length and leaf length/width ratio analysis, plants were grown for 40 days and 60 days. In transgenic cotton, the seed fibres were collected randomly from the natural open bolls and manually measured in fibre length using a ruler. Plant height refers to the length from the cotyledonary node to the main stem growth point of cotton. And were measured two times during cotton growth, the data were sorted out and analysed by GraphPad Prism. Fibre qualities were determined by the cotton fibre quality testing centre, Chinese Ministry of Agriculture (Anyang, Henan).

### Scanning electron microscopy observation

Flowers were harvested at 0 days post‐anthesis, and ovules were dissected from the ovaries under sterile conditions. Ovules were fixation in 2.5% glutaraldehyde overnight at 4 °C. Wash the samples with 1 × PBS buffer (pH7.2) three times and dehydrated with the gradient ethanol (50%, 70%, 85%, 95%, 100%) for 15 min/each. Then the samples were completely dried with liquid CO_2_ by Leica CPD300. After sputtered the ovules with the goldgen for 60s (Leica EM ACE200), fibre initiating cells for ZM 35 and transgenic cotton lines ovules were analysed by SEM (Hitachi SU8100).

### RNA extraction, reverse transcription and real‐time quantitative PCR

Total RNA was isolated from both WT and transgenic cotton lines (*OE‐GhBOP1*, *GhBOP1‐RNAi*) using the RNA Easy Fast Kit (TransGen, Beijing, China). Approximately 1 μg of total RNA was utilized for reverse transcription through the TransScipt First Strand cDNA Synthesis Kit (TransGen, Beijing, China). The SYBR Green Real‐Time PCR Master was employed to prepare the experimental system, and the reactions were conducted on the CFX™ Real‐time PCR systems (Bio‐rad, Foster City, CA, USA). The cotton *GhUb7* gene was used as the reference gene during qRT‐PCR analysis. The data, obtained from three biological replicates and three technical replicates, were analysed using the 2^−ΔΔCT^ method.

### Exogenous BL and BRZ treatment

The brassinolide (BL) was dissolved in ethanol and further diluted to final concentrations of 5, 10, and 50 μM with water. The BR inhibitor, BRZ, was dissolved in dimethylsulfoxide (DMSO) and then diluted to final concentrations of 50, 100, and 250 μM with water. Subsequently, these solutions were applied as sprays to the hypocotyl regions of germinating cotton seeds. A mock control was prepared in DMSO following the same procedure. Following 12 h of dark treatment, the hypocotyl length of transgenic seeds was measured.

### Yeast two‐hybrid assay and direct site mutagenesis

To examine the interaction between GhBOP1 and GhSUMOs (GhSUMO1, GhSUMO2 and GhSUMO3), the full‐length CDS of *GhBOP1* was amplified by PCR and cloned into pGADT7 to create the bait vector. The mature forms of GhSUMOs were similarly amplified and cloned into pGBKT7 to generate the prey vector. The two combination plasmids were then transformed into yeast cells AH109 using the LiAc/PEG methods. To introduce a mutation in the *GhBOP1* sequence, specific primers containing mutation sites were designed, and the mutant sequence was inserted into pGADT7 in place of the original using the same procedure. The monoclonal yeast cells on SD−/−Trp/−Leu medium were subsequently validated by PCR.

### Multiple sequence alignment and phylogenetic analysis

The coding sequence of GhBOP1 and its homologous genes were downloaded from NCBI (www.ncbi.nlm.nih.gov). Sequence alignment was performed using DNAMEN7.0 software, and the phylogenetic analysis was accomplished through the maximum‐likelihood (ML) method in MEGA11.0.

### Co‐immunoprecipitation assay

Co‐immunoprecipitation (Co‐IP) assays were conducted using the *Agrobacterium*‐mediated transient transformation systems in *N. benthamiana* leaves. *Agrobacterium* strains containing recombinant plasmids *GhBOP1‐GFP*, *GhBES1‐HA* and *GhSUMO1‐HA* were mixed in equal proportions and co‐infiltrated into 3–4‐week‐old tobacco leaves. After 48 h, the infiltrated leaves were ground into a fine powder in liquid nitrogen, and dissolved in protein extraction buffer (50 mM Tris–HCl pH7.5, 1 mM EDTA, 150 mM NaCl, 1% Triton‐X100, 1 mM DTT, 1 × Protease Inhibitor Cocktail) and centrifuged at 5180 *g* for 30 min. The resulting supernatants were retained and incubated with GFP beads at 4 °C for 1.5 h. The beads were washed 6–8 times with washing buffer and then boiled with 2× SDS loading buffer at 95 °C for 10 min. The supernatants served as the control (Input). Protein was separated by SDS‐PAGE and immunoblotted with anti‐GFP, anti‐BES1 and anti‐SUMO1 antibodies, respectively.

### Pull‐down assay

GST pull‐down assays were performed using proteins fused with either His or GST tags. GhBOP1 was cloned into the pET28a vector, while the sequences of GhBES1 and GhSUMO1 were cloned into the pGEX6p‐1 vector. Recombinant plasmids were transformed into the *E. coli* strain BL21 through thermal activation. After induction with 100 μM IPTG (isopropylthio‐β‐galactoside) at 16 °C for 5 h, the recombinant proteins were extracted from *E. coli* cells and purified using Ni‐NTA resin or glutathione beads. GST or each GST‐tagged protein was mixed with His‐tagged GhBOP1 proteins in 500 μL pull‐down buffer (10 mM Tris–HCl, 50 mM NaH_2_PO_4_, 300 mM NaCl, 10 mM imidazole, 1 mM PMSF) and incubated at room temperature for 3 h on a tube rotator. His beads were pre‐balanced using a pull‐down buffer to minimize background interference. Approximately 20 μL pre‐balanced beads were added to the reactions. Subsequently, the beads were washed six to eight times with pull‐down buffer and eluted with elution buffer (10 mM Tris–HCl, 50 mM NaH_2_PO_4_, 300 mM NaCl and 20 mM imidazole) The pulled‐down proteins were separated by SDS‐PAGE and detected using anti‐His and anti‐GST antibodies.

### Luciferase complementation imaging (LCI) assay

The LCI assay was conducted following the procedure described previously (Chen *et al*., 2008). The full‐length CDS sequence of GhBOP1 was cloned into pCambia1300‐nLuc, while GhSUMOs were cloned into pCambia1300‐cLuc. The resulting recombinant plasmids were transformed into *Agrobacterium* cells, as described earlier and cultured in lysogeny broth media containing the corresponding antibiotic. After incubating at 28 °C for 12 h, the cells were injected into 3–4‐week‐old tobacco leaves with MMA buffer (10 mM MES, 10 mM MgCl_2_, 200 μM AS, OD_600_ = 1.0). Following 12 h in the dark, the plants were transferred to light conditions for 36 h. Luciferase activity was detected by applying 1 mM substrate to the leaves using the planta imaging system NightShade LB985.

### In vitro SUMOylation

The *in vitro* SUMOylation assay was conducted following the guidelines provided in the SUMOylation Kit (Enzo, Broomfield, CO, USA). The coding regions of GhBOP1 and GhBES1 were cloned into pET28a and pGEX6p‐1, respectively. Then proteins were purified from *E. coli* using Ni‐NTA resin and GST beads. E1 (SAE1b + SAE2), E2 (Ubc9) and SUMO1, obtained from the SUMOylation Kit, were thawed on ice. Approximately 20 ng of E1, 20 ng of E2, 20 ng of SUMO1, 50 ng of GhBOP1, 50 ng of GhBES1, 1 μL of 10 mM Mg‐ATP and 2 μL of SUMOylation buffer was mixed, and ddH_2_O was added to make up a total volume of 20 μL per reaction. Subsequently, all the components were incubated at 37 °C for 1 h, separated using SDS‐PAGE and detected with anti‐SUMO1 or anti‐GST antibodies.

### Cell‐free degradation

The cell‐free degradation assay was performed following the procedure outlined previously (Wang *et al*., 2009). Leaves from 10‐day‐old cotton seedings were collected and ground into a fine powder using liquid nitrogen. Total proteins were extracted in an extraction buffer containing 30 mM Tris–HCl, 10 mM NaCl, 10 mM MgCl_2_, 1 mM PMSF, 4 mM DTT and 10 mM ATP. Protein concentrations were adjusted to an equal level using the 2D‐Kit manuscript for protein quantification. Subsequently, approximately 100 ng of GhBES1‐GST proteins were mixed with 50 μL extracts per reaction. β‐Action antibodies were used as the reference protein in the assay, and exogenous MG132 was used as the negative control. The components were incubated at 22 °C, and each reaction was halted at the indicated times. Protein abundance was assessed using anti‐BES1 for immunoblots, and the bands were quantified using ImageJ software.

### Virus‐induced gene silencing

The procedures for constructing TRV‐related vectors were adapted from a previous report (Liu *et al*., 2004). To generate cotton plants with simultaneous silencing of *GhBES1* and *GhBOP1*, the specific sequences of *GhBES1* and *GhBOP1* were spliced together and cloned into pTRV2e (pYL156) vectors. For the development of *GhBOP1*‐silenced or *GhBES1*‐silenced cotton plants, the TRV: *GhBOP1* and TRV: *GhBES*1 vectors were subsequently constructed. The combined plasmids pTRV1 (pYL192) and pTRV2e (TRV: *GhBOP1*, TRV: *GhBES1*, TRV: *GhBOP1GhBES1*) were transformed into *Agrobacterium* cells. The strains were cultured in LB media and centrifuged at 12 000 rpm for 10 min to collect cells. The cells were then resuspended with MMA buffer and infiltrated into the fully expanded cotyledons of 15 seedings. After 2 weeks, the silencing efficiency was examined by performing qRT‐PCR.

### Accession number

Sequence data for cotton in this study are available in the Cotton Genome Database (www.cottongen.org) and CottonFGD (www.cottofgd.net) under the following accession number: *GhBOP1* (Gh_A09G137700.1), *GhBOP2* (Gh_A01G217800.1), *GhBES1* (Gh_A09G07611), *GhSUMO1* (Gh_A05G316700.1), *GhSUMO2* (Gh_A08G056000.1), *GhSUMO3* (Gh_A05G316700.1), *GhDWF4* (Ghi_A11G10256), *GhCPD* (Ghi_A10G023880.1), *GhACS5* (Ghi_D11G036620.1), *GhPER5* (Ghi_A10G018380.1), *GhNPR1* (Gh_A08G281300.1), GhAP2L (Gh_A08G1156), GhbHLH282 (Gh_D06G0273), GhEXP8 (Gh_A05G028400), Sequence data for other plants in this study can be found at GenBank (www.ncbi.nlm.nih.gov/genbank) and tair (www.arabidopsis.org) under the following accession number: *AtBOP1* (AT3G57130), *AtBOP2* (AT2G41370), *AtNPR1* (AT1G64280), *AtNPR3* (AT5G45110), *AtNPR4* (AT4G19660), *AtCPD* (AT5G05690), *AtPER5* (AT5G17820), *AtDWF4* (AT3G50660), *AtACS5* (At5G65800), *SlBOP1* (NC_015441,3), *SlBOP2* (NC_015447.3), *SlBOP3* (NC_015447.3), *NtBOP1* (AFK30388.1), *LlBOP1* (AGO64649.1), *MtNOOT1* (Q2HW56.1) and *PsCOCH* (G8GTN7.1).

## Author contributions

J.Wu designed the research. B.Wang performed the research, Z.Wang, Ye Tang and N.Zhong analysed the data and results. The manuscript was written by J.Wu and B.Wang.

## Supporting information


**Figure S1** Isolation of GhBOP1 and protein structure analysis.
**Figure S2** Analysis of GhBOP1 expression in transgenic plants shown in Figure 1.
**Figure S3** Comparison of the phenotypes between GhBOP1‐OX transgenic and wild‐type (Col‐0) Arabidopsis plants.
**Figure S4** GhBOP1 interacted with GhBES1.
**Figure S5** Predicted SUMO consensus and SUMO‐interaction motifs (SIMs) in the GhBOP1 protein.
**Figure S6** Sequence alignments of GhBOP1 SIMs among various organisms.
**Figure S7** A propose working model for the mechanism of GhBOP1 regulating GhBES1.
**Figure S8** Predicted SUMO consensus and SUMO‐interaction motifs (SIMs) in the GhBOP1 protein.
**Figure S9** Sequence alignments of GhBOP1 SIMs among various organisms.
**Figure S10** A propose working model for the mechanism of GhBOP1 regulating GhBES1.
**Table S1** The fiber quality of OE‐GhBOP1 and GhBOP1‐RNAi lines and ZM35 growing in Yuncheng, China.

## Data Availability

The data that support the findings of this study are available on request from the corresponding author. The data are not publicly available due to privacy or ethical restrictions.
