# Supplementary material for: Cotton BOP1 mediates SUMOylation of GhBES1 to regulate fibre development and plant architecture
Source: Plant Biotechnol J. 2024 Jul 14;22(11):3054–67. doi: 10.1111/pbi.14428 (PMC11500983; doi:10.1111/pbi.14428)
Supplement: Supplementary file 1 — Figure S1 Isolation of GhBOP1 and protein structure analysis. Figure S2 Analysis of GhBOP1 expression in transgenic plants shown in Figure 1. Figure S3 Comparison of the phenotypes between GhBOP1‐OX transgenic and wild‐type (Col‐0) Arabidopsis plants. Figure S4 GhBOP1 interacted with GhBES1. Figure S5 Predicted SUMO consensus and SUMO‐interaction motifs (SIMs) in the GhBOP1 protein. Figure S6 Sequence alignments of GhBOP1 SIMs among various organisms. Figure S7 A propose working model for the mechanism of GhBOP1 regulating GhBES1. Figure S8 Predicted SUMO consensus and SUMO‐interaction motifs (SIMs) in the GhBOP1 protein. Figure S9 Sequence alignments of GhBOP1 SIMs among various organisms. Figure S10 A propose working model for the mechanism of GhBOP1 regulating GhBES1. Table S1 The fiber quality of OE‐GhBOP1 and GhBOP1‐RNAi lines and ZM35 growing in Yuncheng, China. [file PBI-22-3054-s001.docx]

Supplementary Information for

**Cotton *BOP1* mediates SUMOylation of GhBES1 to regulate fiber development and plant architecture**

Bingting.wang *et al*

*Corresponding author. Email: jiahe.wu@im.ac.cn

**This file includes:**

Figs. S1 to S10.

Table S1.

Supplementary Figure1. Isolation of GhBOP1 and protein structure analysis. (a) an unrooted phylogenetic tree of proteins predicted to contain BTB/POZ domains in Arabidopsis, *Gossypium hirsutum,* and *Solanum lycopersicum*. A maximum likelihood tree was generated with Mega 11.0. Bootstrap values were calculated from 1000 replicates. (b) Conserved domains in the GhBOP1 protein. The light grey box represents the BTB/POZ domain. Dark gray boxes show ankyrin-repeat (ANK). (c) Alignment of the predicted amino acid sequence of GhBOP1 with AtBOP1 and GhBOP2 proteins. The BTB/POZ domain and ankyrin repeats are marked.


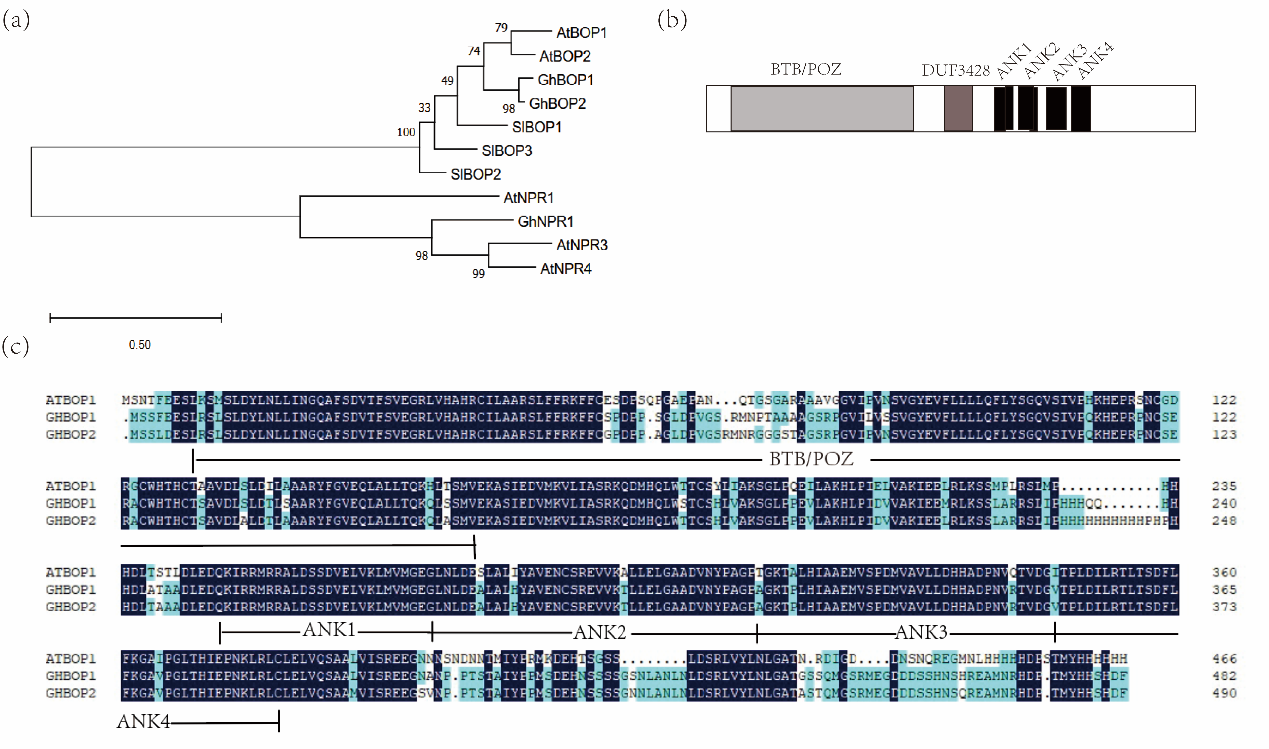


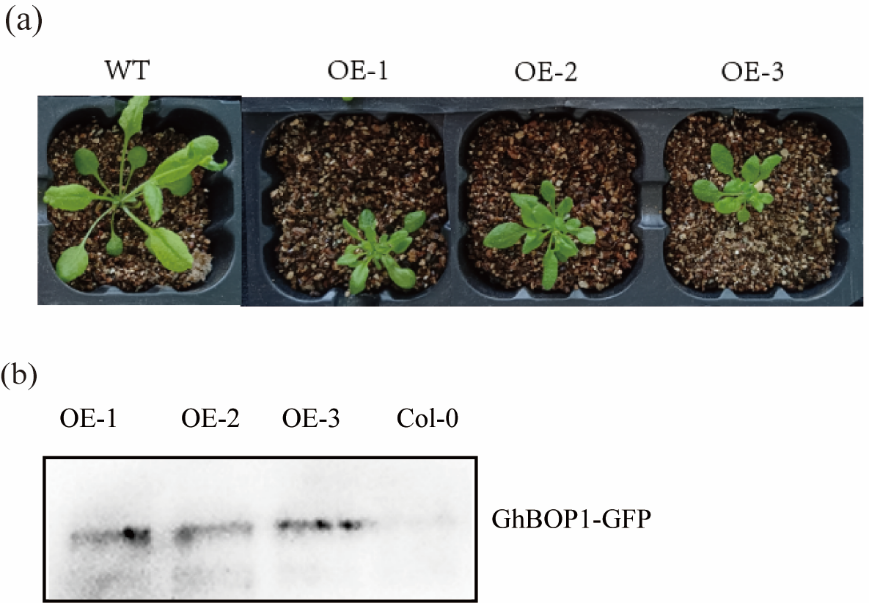


Supplementary Figure 2. Analysis of GhBOP1 expression in transgenic plants shown in Figure 1. (a) Overexpressing *GhBOP1* showed a BR-deficient phenotype. Wild type (WT) and GhBOP1-OX plants were grown in soil under long-day conditions for 12 days. (b) Western blot analysis of protein expression of GhBOP1 in T3 transgenic Arabidopsis GhBOP1-OX. Anti-GFP was used to detect the GhBOP1 in total protein.


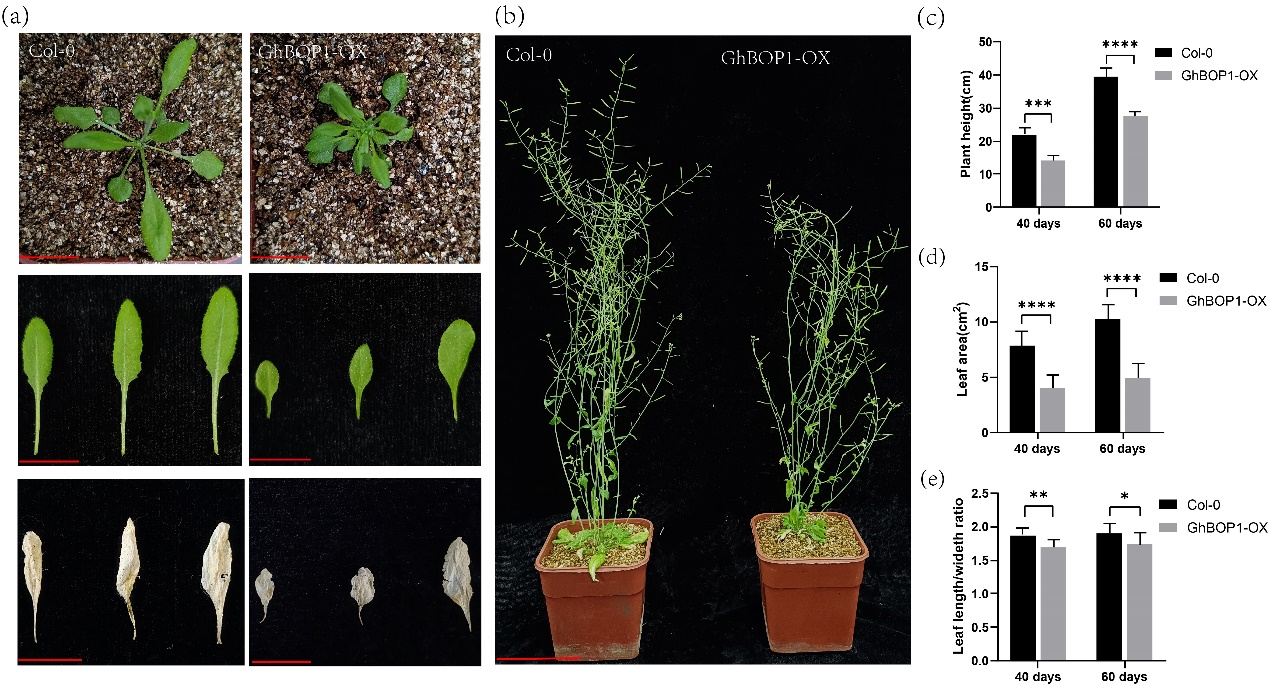


Supplementay Figure 3. Comparison of the phenotypes between GhBOP1-OX transgenic and wild-type (Col-0) Arabidopsis plants. (a) Rosettes and the fifth leaves of 3-week-old plants (upper and middle) and the fifth leaves of 6-week-old plants (lower). scale bars: 2 cm. (b) and (c), Plant height. Scale bars: 8cm. The plants were grown on 1/2 MS medium under long-day conditions (16-hours light/8-hours dark cycles) for 10 days and in soil for 4 weeks. More than 7 samples were tested. Students’ t-test, * *P* <0.05.(d), Leaf area of the fifth leaves. (e) Ratio of leaf length/width of the fifth leaves. More than 9 samples were randomly measured. All data were analysed using students’ *t*-test with GrapdPad Prism. *****P*<0.0001,****P*<0.001,***P*<0.01,**P*<0.05, ns *P*>0.05

Supplementary Figure 4 Relative expression level of BR responsive genes in 30-day old GhBOP1-OX plants. The expression levels in wild type (Col-0) were defined as “1”. Data were means of three biological repeats. **P*<0.05, **P*<0.01.

Supplementary Figure 5 The expression levels of BR related genes involved in cotton fiber elongation in 10 DPA fibers of OE-GhBOP1, WT, and GhBOP1-RNAi lines. The expression levels in wild type (ZM35) were defined as “1”. Data were means of three biological repeats. Student’s *t*-test, **P*<0.05, **P*<0.01,****P*<0.001.


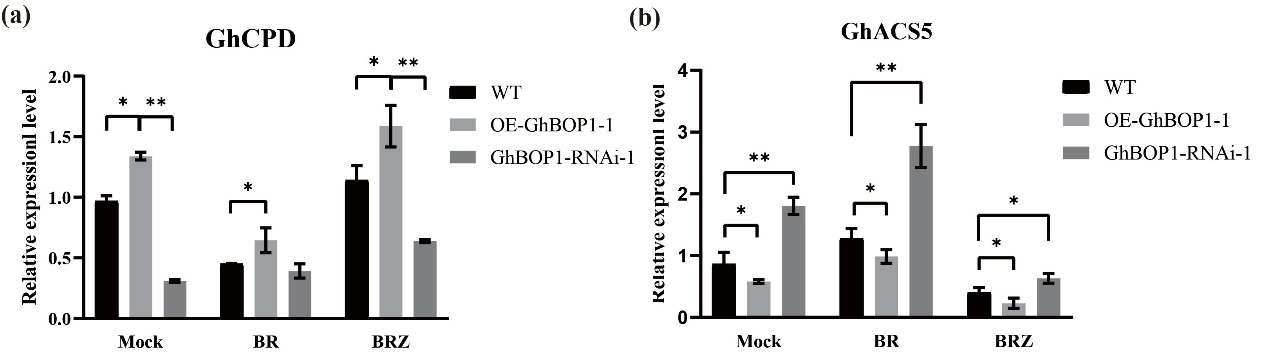


Supplementary Figure 6 (a) The expression level of GhCPD in WT, OE-GhBOP1, and GhBOP1-RNAi seedings after 3h being treated with DMSO, 50nM BRZ, or 250nM-BL. (b) The expression level of GhACS5 in WT, OE-GhBOP1, and GhBOP1-RNAi seedings after 3h being treated with DMSO, 50nM BRZ, or 250nM-BL. Data were means of three biological repeats. Student’s *t*-test, **P*<0.05, **P*<0.01,****P*<0.001.

­


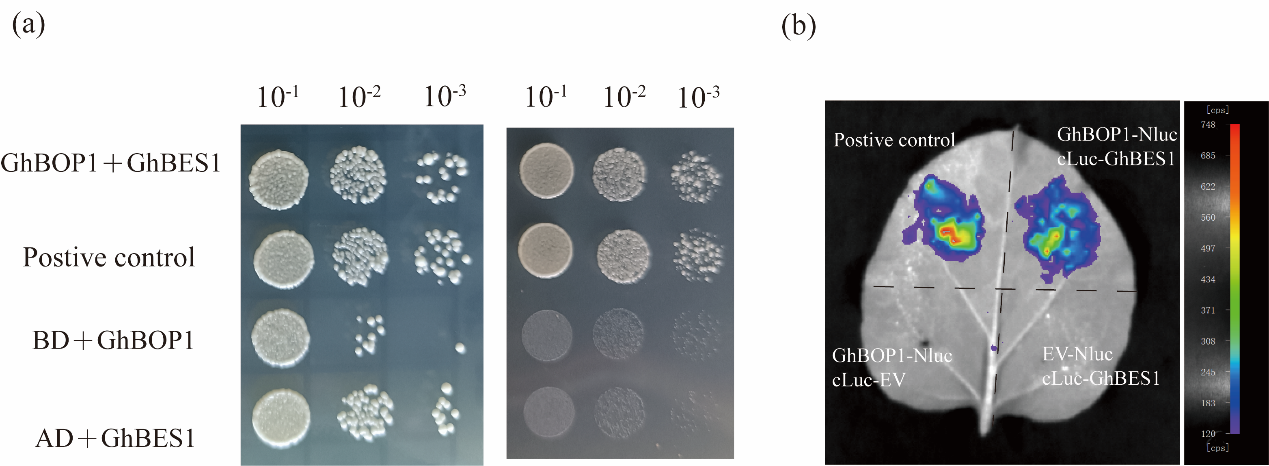


Supplementary Figure 7 GhBOP1 interacted with GhBES1.(a), Yeast two-hybrid. The same concentration of AH109 yeast cells carrying AD-GhBOP1 and BD-GhBES1 proteins were spotted on SD-LW and SD-LWHA. The photos were taken after 3 days at 28℃. (b) The luciferase complementary assays. The GhBOP1 and GhBES1 proteins were fused to either the C- or N-terminal half of luciferase (cLuc or nLuc), and transiently expressed in *N. benthamiana*. The luminescence signal was detected by applying 1 mM luciferin after 60 h of agroinfiltration

_
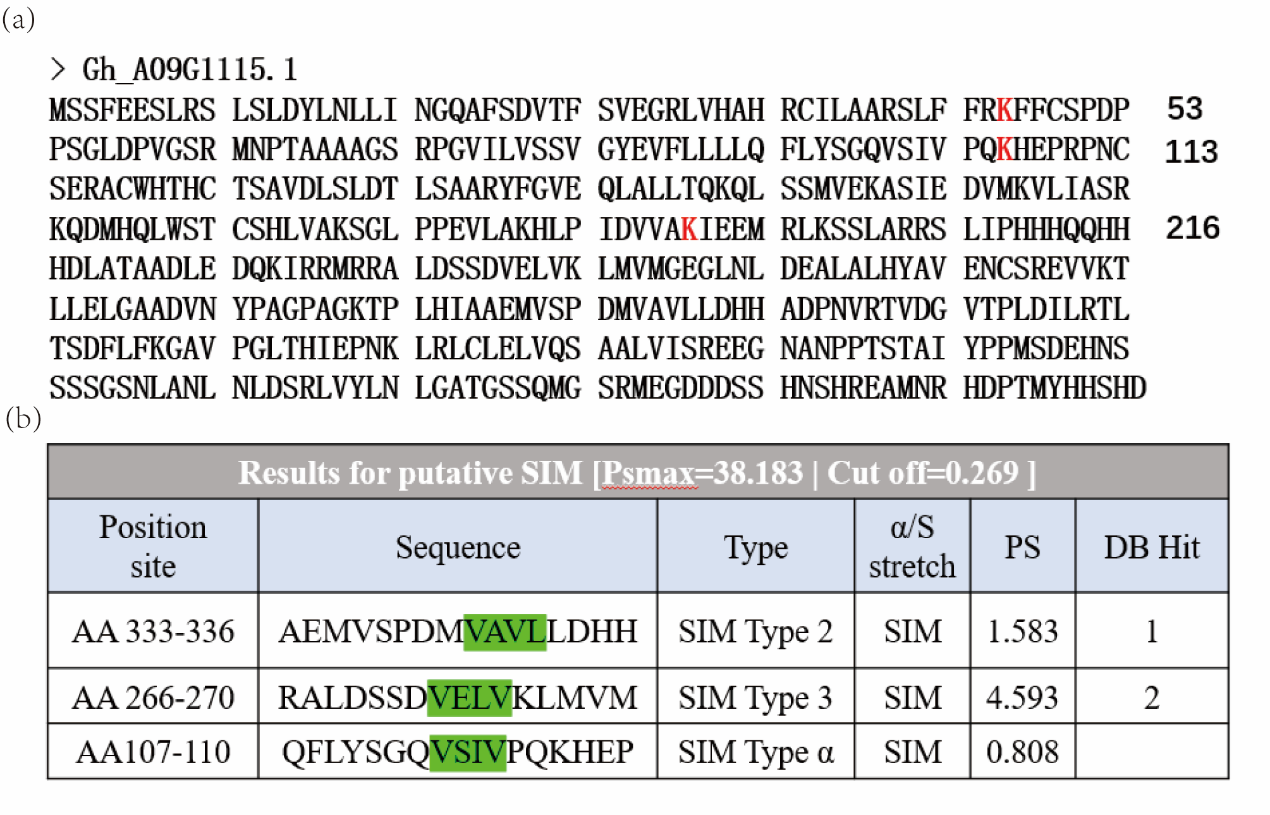
_

Supplementary Figure 8 Predicted SUMO consensus and SUMO-Interaction Motifs (SIMs) in the GhBOP1 protein. The SUMO target lysines were shown in red (K53, K113, K216). The table resumes the results for putative SIMs. The SIMs were labeled in green. Consensus type 2, [V/L]-[X]-[V/L]-[V/L]. Consensus type3, [V/I/L]-[D/E]-[V/I/L]-[V/I/L]. Consensus type α, [V/I]-[X]-[V/I]-[V/I]. α/S stretch, a potential acidic/serine stretch. PS, predictive scores. DB Hit, database hit matching the query sequence.

_
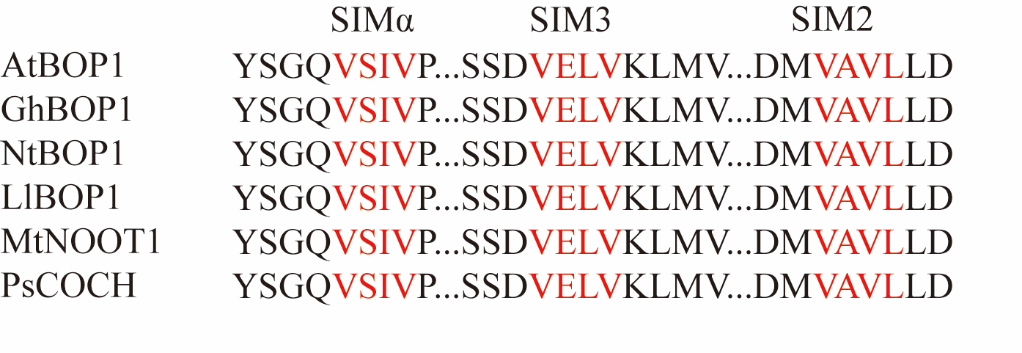
_

Supplementary Figure 9 Sequence alignments of GhBOP1 SIMs among various organisms.


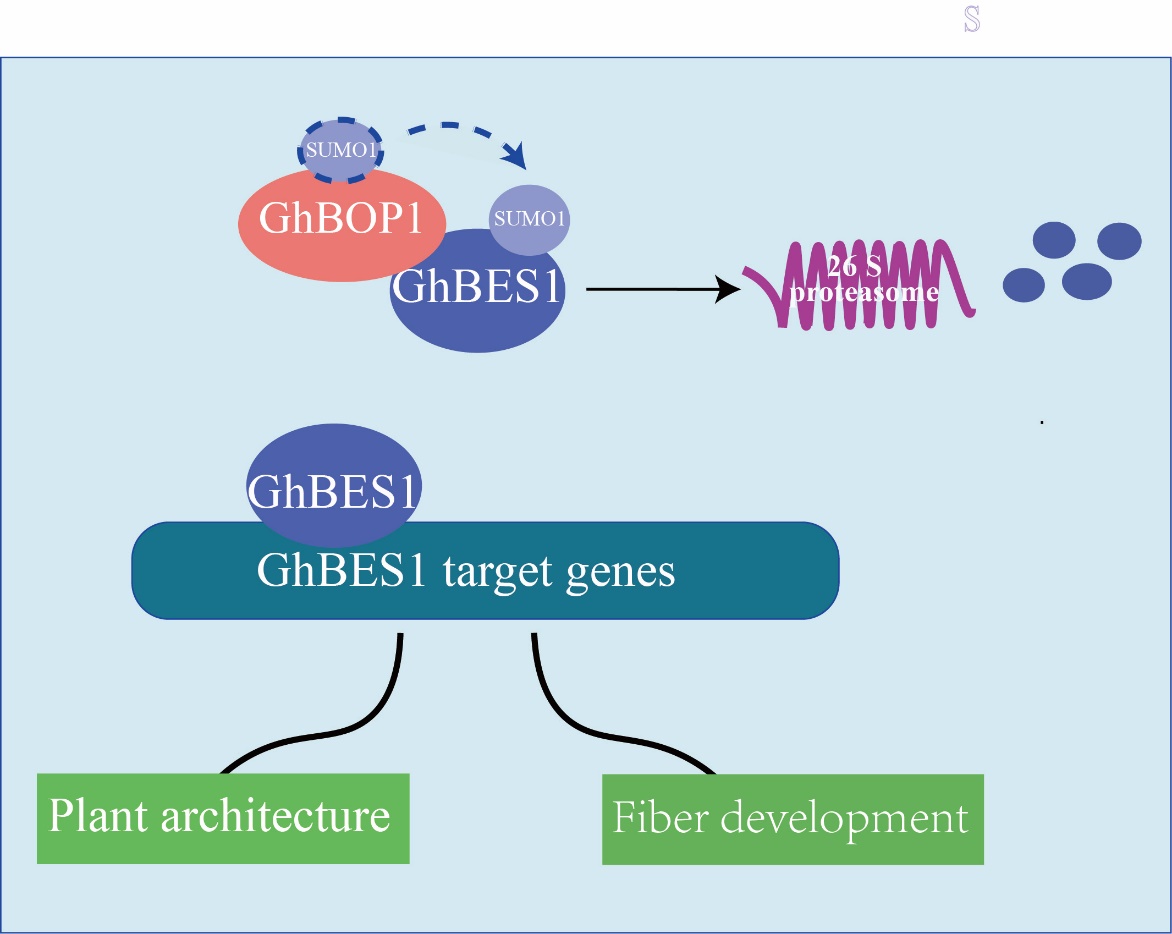


Supplementary Figure 10 A propose working model for the mechanism of GhBOP1 regualting GhBES1. while GhBOP1 interacted with GhBES1 and mediated its SUMOylation to promote its degradation, thus influencing the downstream target genes expression and regulating the plant growth and cotton fiber development. The Dotted line represented the substrate SUMO1 protein transfer to the GhBES1 protein.

Supplemental Table 1. The fiber quality of OE-GhBOP1 and GhBOP1-RNAi lines and ZM35 growing in Yuncheng, China.

| Line | Average length of upper quartile fibers (mm) | uniformity index (%) | breaking tenacity (cN•tex^-1^) | micronaire  value | breaking elongation  (%) | lint percentage  (%) |
| --- | --- | --- | --- | --- | --- | --- |
| ZM35 | 28.8±0.30 | 85.0±0.15 | 28.6±0.30 | 3.9±0.10 | 6.5±0.45 | 39.8±2.4 |
| OE-GhBOP1-1 | 26.8±0.15* | 83.3±0.40 | 27.6±0.50 | 4.2±0.25 | 6.8±0.55 | 38.6±1.5 |
| OE-GhBOP1-2 | 27.0±0.25* | 84.1±0.60 | 30.8±0.25* | 4.1±0.65 | 7.0±0.45* | 41.8±1.8 |
| GhBOP1-RNAi-1 | 29.4±0.35 | 85.4±0.60 | 29.8±0.50* | 4.3±0.20 | 6.4±0.40 | 39.7±2.4 |
| GhBOP1-RNAi-2 | 28.7±0.35 | 85.2±0.75 | 30.7±0.30* | 4.5±0.40 | 5.6±0.30* | 39.5±4.1 |

The data was analysed by Graphpad Prism using students *t-*test. **P*<0.05.
